# *Prevotella* contributes to individual response of FOLFOX in colon cancer

**DOI:** 10.1002/ctm2.512

**Published:** 2021-09-26

**Authors:** Xiao‐Ying Hou, Pei Zhang, Hong‐Zhi Du, Yi‐Qiao Gao, Rui‐Qi Sun, Si‐Yuan Qin, Yuan Tian, Jing Li, Yu‐Xin Zhang, Wei‐Hua Chu, Zun‐Jian Zhang, Feng‐Guo Xu

**Affiliations:** ^1^ Key Laboratory of Drug Quality Control and Pharmacovigilance (Ministry of Education) State Key Laboratory of Natural Medicine China Pharmaceutical University Nanjing China; ^2^ School of Pharmacy Hubei University of Chinese Medicine Wuhan China; ^3^ China Pharmaceutical University School of Life Science and Technology Nanjing P. R. China; ^4^ the Affiliated Hospital of Nanjing University Medical School Nanjing Drum Tower Hospital Nanjing China

Dear Editor:

FOLFOX, the combination of 5‐FU, calcium folinate, and oxaliplatin, is the first‐line chemotherapy in advanced colorectal cancer.^1^ However, only 30–50% of patients could benefit from FOLFOX treatment, and this distinct individualized drug response seriously restricted its application.^2^, ^3^ Increasing evidence has shown that gut microbiota is involved in modulating chemotherapy efficacy,^4^, ^5^ while whether inherent heterogeneity of the gut microbiota is contributing to the individual response of FOLFOX remains elusive.

First, a CT‐26 colon cancer xenograft mouse model was constructed and treated with FOLFOX (Figure 1A). As a result, the tumor development was significantly inhibited starting from day 5, accompanied by decreased body weight (Figure 1B–D). In accordance with clinical practice, large individualized drug efficacy of FOLFOX was observed (Figure S1). Based on the relative tumor volume (RTV) and Ki67 assessment, the mice treated with FOLFOX were divided into two groups: FOLFOX sensitive (S, *n* = 8) and FOLFOX nonsensitive animals (NS, *n* = 9) (Table S1). As shown in Figure 1E and F, there is no significant difference in body weight or tumor volume between the S and NS groups before FOLFOX administration. Importantly, a remarkable difference in the antitumor effect was observed after FOLFOX treatment (Figure 1G–I).

**FIGURE 1:**
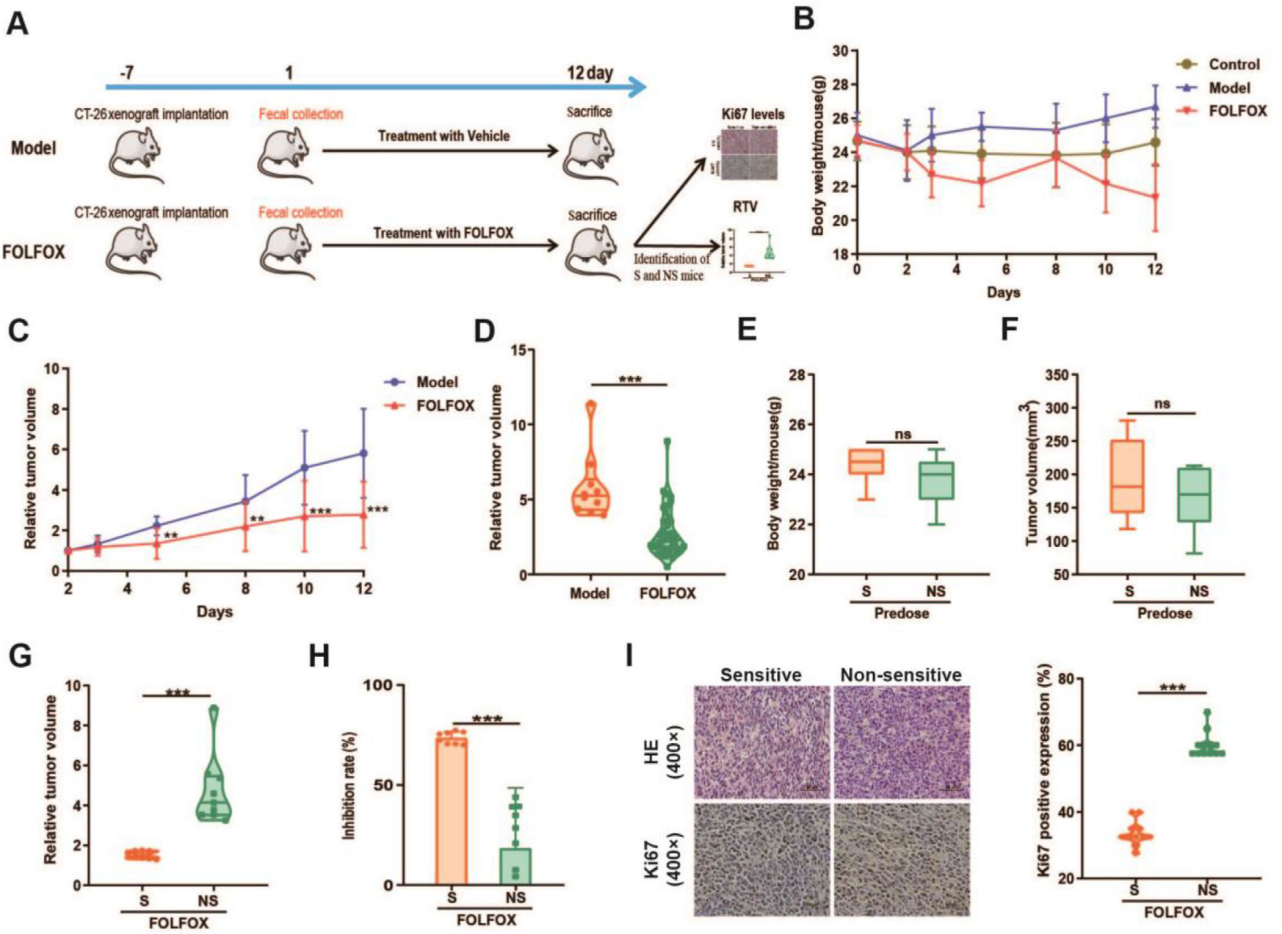
Recognition of S and NS individuals after FOLFOX treatment. (A) Schematic of the recognition of individual difference after FOLFOX treatment in CT‐26 colon cancer xenograft model. (B) The effects of FOLFOX on the body weight of tumor‐bearing mice. (C, D) FOLFOX treatment could inhibit tumor development. There is no obvious difference in (E) body weight and (F) tumor volume in S and NS groups before FOLFOX administration. (G) RTV, (H) inhibition rate, and (I) Ki67 levels of the S (*n* = 8) and NS (*n* = 9) groups at the end of the experiment (day 12). Data were expressed as mean ± SD. It was considered statistically significant when *p* < 0.05, *p < 0.05, **p < 0.01, ***p < 0.001

To determine whether gut bacteria contribute to the individualized effect of FOLFOX, we performed a 16S rRNA gene sequencing analysis on predose fecal samples from S and NS groups. While no significant difference was observed between the two groups, the α‐diversity indices, including Chao1 (reflecting microbial community richness), Shannon, and Simpson (reflecting microbial community diversity), were relatively higher in the S group (Figure 2A–C). Meanwhile, partial least‐squares discriminant analysis (PLS‐DA) of β‐diversity suggests that the compositions of bacterial communities differed significantly between the sensitive and nonsensitive individuals (Figure S2). Importantly, the gut bacterial composition was prominently different at phylum and genus levels between the two groups (Figure 2D, E). In particular, the relative abundance of *Staphylococcus*, *Jeotgalicoccus*, and *Sphingomonas* was significantly increased in the S group, whereas *Prevotella* was higher in NS individuals (Figure 2F–I).

**FIGURE 2:**
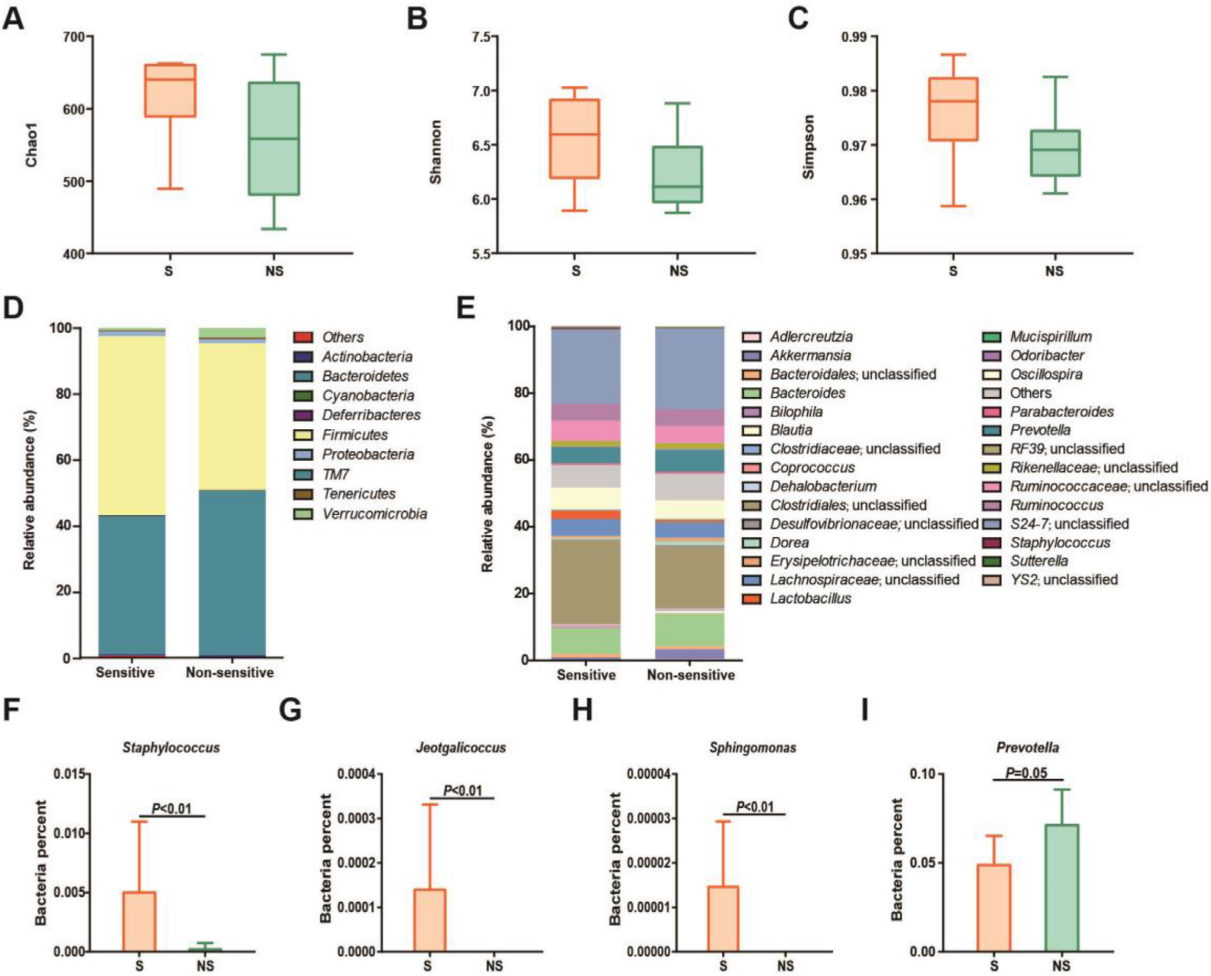
Screening of potential bacterial genus contributing to individualized FOLFOX response. (A–C) The α‐diversity indexes of Chao1 (*p *= 0.1729), Shannon (*p *= 0.0809), and Simpson (*p *= 0.1177) of gut microbiota between S and NS groups. Taxonomic distributions of bacteria from S and NS groups at (D) the phylum level and (E) genus level. (F–I) Comparison of relative abundance of significantly changed bacterial species between S and NS mice

Next, we evaluated whether the four bacterial genera could modulate the sensitivity of FOLFOX in tumor‐bearing mice. Referring to previous reports,^6^, ^7^ ABX (an oral antibiotics cocktail consisted of ampicillin, metronidazole, neomycin sulfate, and vancomycin) pretreatment integrated with bacterial gavage was applied to realize bacterial colonization on the CT‐26 xenograft mouse model (Figure S3). The relative level of each bacterial genus increased after colonization according to qPCR assay (Figure S4), demonstrating the success of the bacterial transplantation. We then investigated the effect of aerobic bacteria (i.e., *Staphylococcus*, *Jeotgalicoccus*, and *Sphingomonas*) and anaerobic bacteria (i.e., *Prevotella*) transplantation on FOLFOX efficacy separately. As a result, ABX pretreatment alleviated the decrease of body weight caused by FOLFOX (Figures S5A and 3A). Moreover, the anticancer effect was enhanced with FOLFOX and ABX combination compared to FOLFOX alone, manifested by increased tumor inhibition rates from 45.25% to 75.39% (Figure S5D) and 33.35% to 50.88% (Figure 3D) for aerobic and anaerobic bacterial transplantation, respectively. The colonization of *Staphylococcus*, *Jeotgalicoccus*, or *Sphingomonas* had no influence on the therapeutic effect of FOLFOX (Figure S5). Notably, the efficacy of FOLFOX was significantly suppressed with the colonization of *Prevotella* (*p *< 0.05), indicated by a reduced tumor inhibition rate from 50.88% to 27.04% (Figure 3B–D). In addition, the percentage of Ki67 positive cells which reflects the proliferation of tumor significantly increased with *Prevotella* colonization (Figure 3E). In a word, these results suggest a negative role of *Prevotella* in FOLFOX treatment.

**FIGURE 3:**
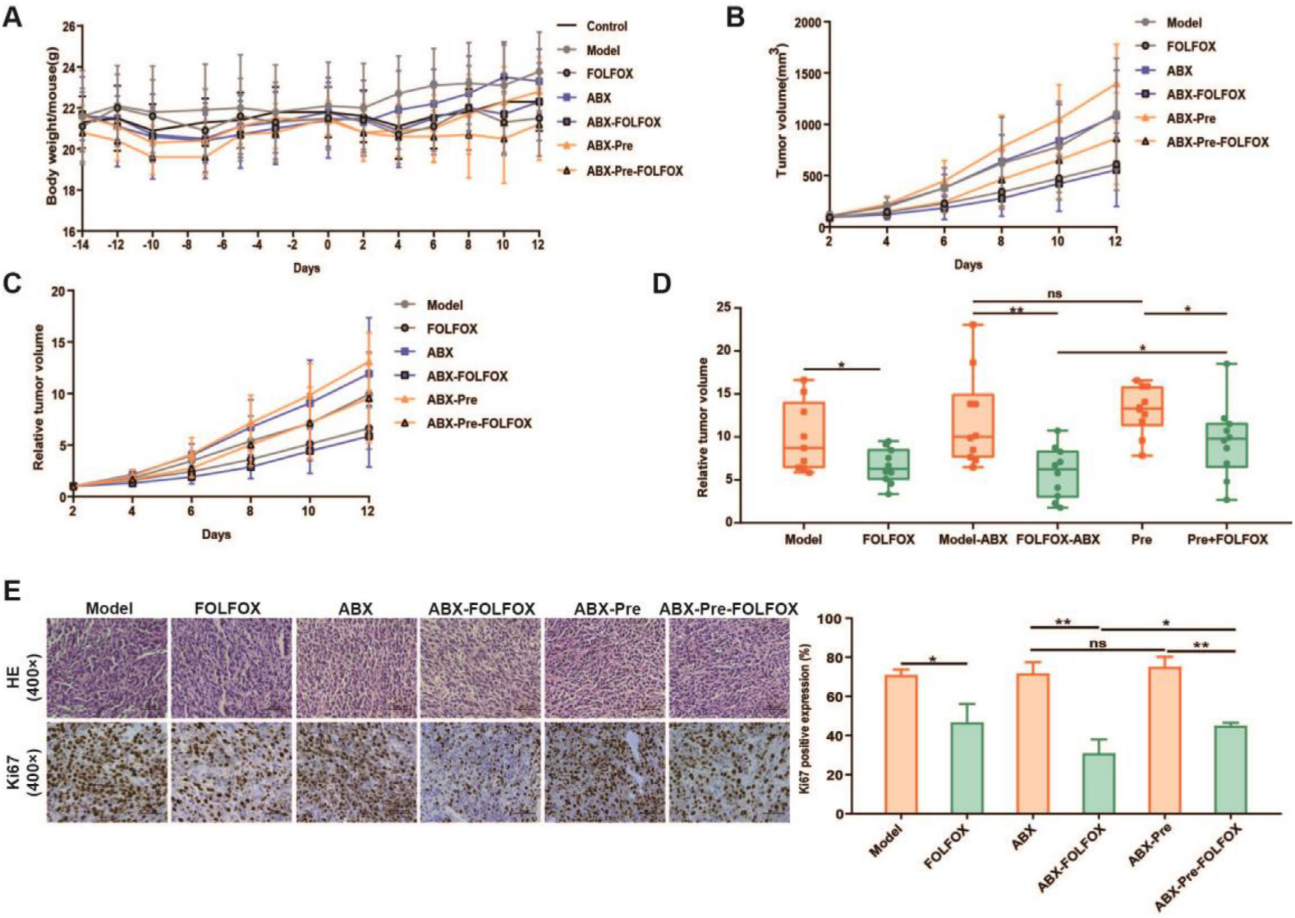
Effects of anaerobic bacteria transplantation on FOLFOX efficacy. (A) Change of mice body weight across the experiment. Tumor volume (B) and relative tumor volume (C, D) were recorded throughout the experiment. Inhibition rate: Model versus FOLFOX (33.35%); ABX versus ABX‐FOLFOX (50.88%); ABX‐Pre versus ABX‐Pre‐FOLFOX (27.04%). (E) Ki67 levels were compared in the Model versus FOLFOX group, ABX versus ABX‐FOLFOX group, ABX‐Pre versus ABX‐Pre‐FOLFOX group. Data were expressed as mean ± SD. It was considered statistically significant when *p* < 0.05, *p < 0.05, **p < 0.01

To explore the potential microbiota–metabolite axis responsible for the individualized sensitivity of FOLFOX, predose fecal samples from S and NS groups were measured by a nontarget metabolomics approach. OPLS‐DA models were constructed to explore metabolic differences between S and NS groups (Figure S6). As a result, 20 differential metabolites were obtained (Figure S7 and Table S2). Among them, 3‐Oxocholic acid (3‐Oxo) and N‐acetyl‐L‐methionine were most significantly altered with a fold change of 33.83 and 12.56, respectively. We then analyzed the correlations between *Prevotella* and all the differential metabolites by Spearman correlation analysis. As shown in Figures 4A–C and S8, the levels of indole‐3‐carboxylic acid, 3‐Oxo, and phenylalanyl‐asparagine were positively correlated with the abundance of *Prevotella* (*p *< 0.05).

**FIGURE 4:**
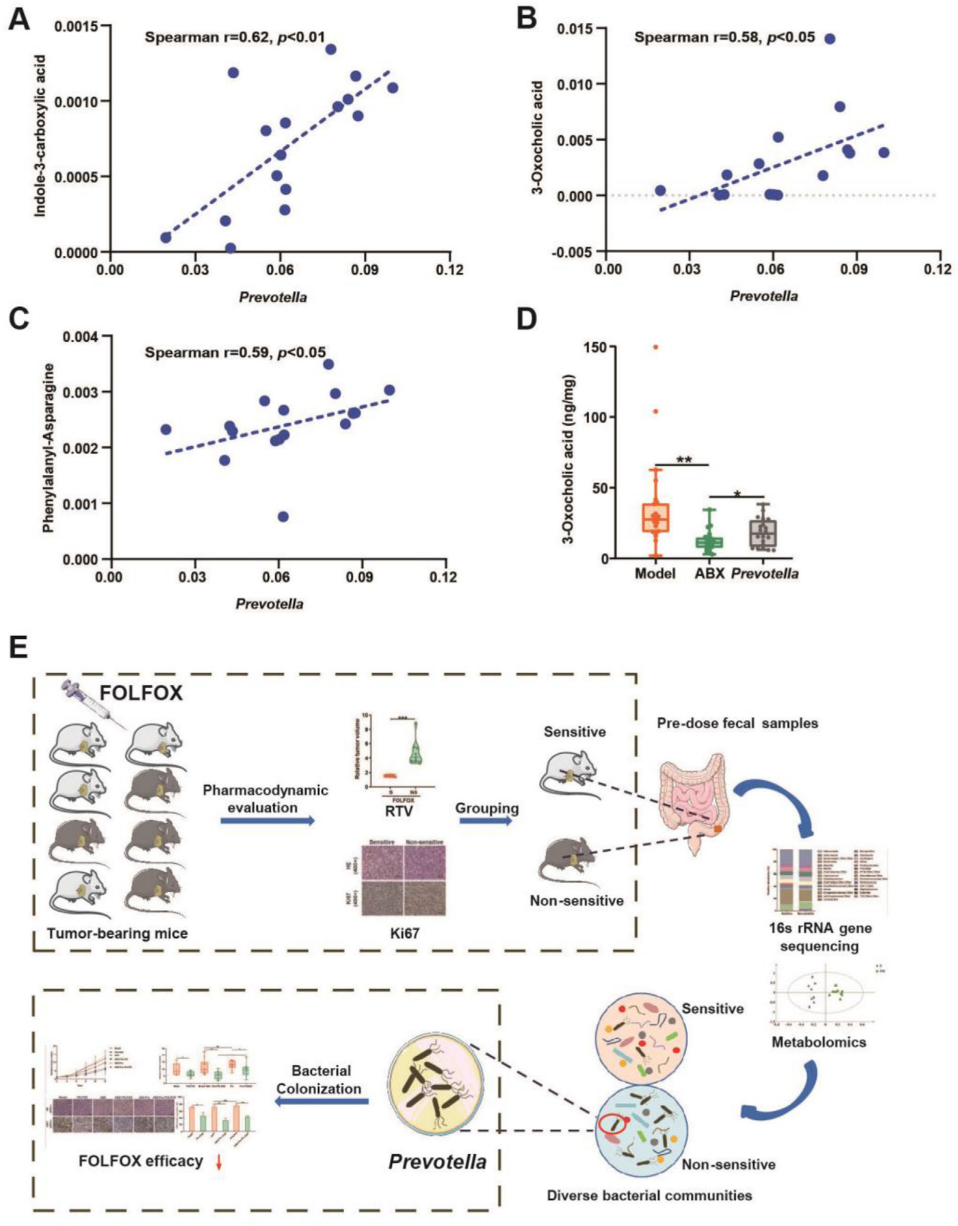
Metabolomics explore the key metabolite responsible for gut microbiota‐mediated FOLFOX efficacy. Relative abundance of (A) indole‐3‐carboxylic acid, (B) 3‐oxocholic acid, and (C) phenylalanyl‐asparagine was significantly associated with the level of *prevotella* as measured by Spearman's correlations analysis. (D) Concentrations of 3‐Oxo in fecal samples from Model (*n* = 20), ABX (*n* = 20) and *Prevotella* (*n* = 20) group, respectively. (E) Workflow of the study. Data were expressed as mean ± SD. It was considered statistically significant when *p* < 0.05, *p < 0.05, **p < 0.01

With the above results and considering 3‐Oxo is a gut microbiota metabolized product from cholic acid (CA, Figure S9), we further explored the relationship between 3‐Oxo and *Prevotella*. First, the absolute concentration of bile acids involved in the 3‐Oxo pathway in fecal samples after *Prevotella* colonization was determined. The results suggest that ABX pretreatment inhibited the biotransformation from CA to 3‐Oxo, and this was significantly rescued by *Prevotella* colonization (Figures S10 and 4D). Transforming CA into 3‐Oxo is a reversible reaction catalyzed by 3α hydroxysteroid dehydrogenases (3α‐HSDH) (Figure S9). Therefore, we utilized bioinformatics analysis to find bacterial species that contain 3α‐HSDH paralogs (Table S3). *Prevotella buccae* was focused with a 45% sequence identity. Then, it was confirmed that both *P. buccae* and 3α‐HSDH were positively detected in cultured *Prevotella*, demonstrating *Prevotella* does encode 3α‐HSDH that is responsible for the transformation of CA to 3‐Oxo. Moreover, the relative abundance of *P. buccae* also obviously increased in the *Prevotella* colonization group (Figure S11). All these results suggest the significant role of 3‐Oxo in *Prevotella*‐mediated individualized efficacy of FOLFOX. To further confirm this, we investigated the effect of 3‐Oxo on colon cancer cells and the anticancer effect of FOLFOX. The preliminary exploration indicated that 3‐Oxo could significantly improve the expression of P‐EGFR/P‐ERK/c‐MYC and LOX, reverse the anticancer effect of FOLFOX, promote cancer metastasis, and induce the secretion of inflammatory factors (Figures S12 and 13).

In this study, we found *Prevotella* and 3‐Oxo could promote malignant progression of colon cancer, reverse the anticancer effect of FOLFOX, and might be responsible for the individualized FOLFOX efficacy (Figure 4E). However, further experiments conducted in germ‐free mice and verifications in clinical practice are needed. Meanwhile, our experiments were performed at the genus level due to the limited precision of 16S rRNA gene sequencing analysis. In future explorations, the screening of bacterial strain or strain combinations responsible for the individualized FOLFOX efficacy in a larger scale of animals is of great importance. In conclusion, the critical role of gut microbiota on the individualized anticancer effect of FOLFOX was revealed in our study. *Prevotella* is potentially a novel predictive biomarker of FOLFOX response as well as a therapeutic target for colon cancer.

## CONFLICT OF INTEREST

The authors declare that there is no conflict of interest.

## ETHICS STATEMENT

The study was conducted in accordance with the standards established by the Experimental Animal Care Commission in China Pharmaceutical University (License No: SYXK 2018‐0019).

## AUTHOR CONTRIBUTIONS

Xiao‐Ying Hou, Wei‐Hua Chu, and Yi‐Qiao Gao designed the experiments. Xiao‐Ying Hou, Hong‐Zhi Du, Rui‐Qi Sun, Si‐Yuan Qin, and Jing Li performed the experiment and data analysis. Xiao‐Ying Hou wrote and commented the manuscript. Yuan Tian assisted instrument operation and maintenance. Yu‐Xin Zhang, Pei Zhang, Zun‐Jian Zhang, and Feng‐Guo Xu designed the study, commented the manuscript, and supervised the study. All authors read and approved the final manuscript.

## DATA AVAILABILITY STATEMENT

The raw data for 16S rRNA gene sequence have been deposited in the NCBI BioProject database (https://www.ncbi.nlm.nih.gov/bioproject/) under accession number PRJNA679751.

## Supporting information

Supporting information
