# Supplementary material for: Prevotella contributes to individual response of FOLFOX in colon cancer
Source: Clin Transl Med. 2021 Sep 26;11(9):e512. doi: 10.1002/ctm2.512 (PMC8473639; doi:10.1002/ctm2.512)
Supplement: Supplementary file 1 — Supporting information [file CTM2-11-e512-s001.docx]

**Supplementary Information for**

***Prevotella* Contributes to Individual Response of FOLFOX in Colon Cancer**

**1. Supplementary Material and Methods**

***1.1. Chemicals and Reagents***

5-FU Injection was obtained from SunRise (Shanghai, China), Calcium Folinate Injection and Oxaliplatin Injection were obtained from Aosaikang (Jiangsu, China). FOLFOX Injection was prepared according to the existing studies ^1, 2^. Ampicillin (MB1507), Metronidazole (MB2200), Neomycin sulfate (MB1716) and Vancomycin (MB1260) were purchased from Meilunbio (Dalian, China). O-Methoxyamine hydrochloride, N-methyl-N-trifluoroacetamide (MSTFA), Deoxycholic acid (DCA, 98%) was purchased from Sigma-Aldrich (St.Louis, MO, USA). Cholic acid (CA, 98%), Ursodeoxycholic acid, (UDCA, 98%) and Cortisone acetate (IS, 97%) was purchased from J&K Scientific Ltd. (Beijing, China). 3-dehydrocholic acid (3-Oxo, 97%) was purchased from Toronto Research Chemicals (Toronto, Canada).

***1.2. Cell Culture***

Mice colon cancer cell line CT-26 was obtained from the Cell Bank of the Institute of Biochemistry and Cell Biology, Chinese Academy of Sciences (Shanghai, China), the cells were cultured in RPMI-1640 (Gibco, Grand Island, USA) with 10% Fetal Bovine Serum (Gibco). THP-1 cell line was obtained from American Type Culture Collection (ATCC, Manassas, USA), the cells were cultured in RPMI-1640 (Gibco) supplied with 10% Fetal Bovine Serum (Gibco) coupled with 1×HEPES buffer (Boster, Wuhan, China). All the cells were incubated at 37 °C in a humidified atmosphere with 5% CO_2_.

THP-1 monocytes were seeded in 24-well plates and stimulated by 100 ng/mL phorbol 12-myristate 13-acetate (PMA; Sigma, St. Louis, USA) for 48 h to differentiate into macrophages. Then, the medium was replaced by fresh medium with specific concentrations of 3-Oxo for 4h. The culture medium supernatant was collected for enzyme-linked immunosorbent assays.

***1.3. Colon cancer xenograft model and samples collection***

Five to six-week-old male BALB/c mice (18 - 22 g) were provided by the Beijing Vital River Laboratory Animal Technology Co. Ltd. (Zhejiang, China) with the permission number SCXK (Zhe) 2019-0001. All the mice were housed in temperature-controlled environment (24±2 °C) under a 12/12 h-dark/light cycle.

200 μL CT-26 cell suspension (about 10^6^ cells) was subcutaneous injected into the flank of mice ^3^. When tumor volume reached to about 100 mm^3^, mice were randomly divided into control group (n=10), model group (n=10) and FOLFOX group (n=40) (day 0). Fecal samples were collected on day 1 before FOLFOX treatment. On the day 2, FOLFOX (6 mg/kg oxaliplatin followed by 50 mg/kg 5-FU and 90 mg/kg calcium folinate in 2 hours) was intraperitoneal (i.p.) administrated to FOLFOX group on a weekly basis (day 2&9) ^1, 2^. Model group was treated with the same vehicle (Figure. 1A). Control group were normally feed without any treatment. Tumor volume was monitored by a vernier caliper throughout the experiment. All the mice were sacrificed at day 12, and tumors were removed and processed for immunohistochemistry analysis.

Tumor volume (TV), Relative tumor volume (RTV) and inhibition rate were calculated by the following formulas:

TV (mm^3^) = A/2 × B^2^, where A represents the longest diameter of tumor, and B represents the shortest diameter;

RTV = V_t_ / V_0_, where V_0_ represents the tumor volume of day 2 (the day of FOLFOX first administration), V_t_ represents the tumor volume of day t;

Inhibition rate (%) = (RTV_m_ - RTV_t_) / RTV_m_ × 100%, where RTV_m_ represents the RTV of model group, and RTV_t_ represents the RTV of treatment group.

***1.4. Histopathology***

Tumor tissues were formalin fixed and paraffin embedded. Sections were then subjected for hematoxylin and eosin (HE) staining and immunohistochemistry as previously reported ^4^. Ki-67 is a nuclear protein expressed in proliferating cells, which has been confirmed correlating with the therapeutic outcomes of cancer patients and applied as a critical tumor pathological grade and prognostic factor in many cancers ^5^. In the current study, tumor proliferation status was assessed by the percentage of positively Ki67 stained cells. Scoring was defined as the proportion of Ki67 positively stained cells in total tumor cells (at least 1000 cells) with 400 fold magnification ^6^.

***1.5. Identification of FOLFOX Sensitive (S) and Non-Sensitive (NS) individuals***

Pharmacodynamics evaluation includes two aspects: RTV and Ki67 levels. FOLFOX treatment mice (n=37) were ranked by RTV and Ki67 levels, respectively. Mice with the lowest level of RTV (30%) were recognized as S1, and the highest RTV (30%) as NS1. Meanwhile, mice with the lowest Ki67 level (30%) were recognized as S2, and the highest Ki67 level (30%) as NS2. The mice belonging to both S1 and S2 were defined as FOLFOX sensitive individuals (group S), and those existed in both NS1 and NS2 were recognized as FOLFOX non-sensitive (group NS).

***1.6. 16S rRNA gene sequencing analysis***

Total bacterial genome DNA was isolated from fecal with Stool Genomic DNA Kit (CWBIO, Beijing, China) according to the manufacturer’s instructions. The DNA concentrations were detected using NanoDrop 2000 (Thermo Fisher Scientific, Waltham, USA) (Fecal sample of F11 was absent due to unsatisfied DNA concentration). After checked by 1% agarose gels electrophoresis for integrity, PCR amplification was performed spanning the V3-V4 hypervariable regions of the bacteria 16S rRNA gene and sequenced on Illumina Hiseq 2500 platform (Illumina, San Diego, USA) ^7, 8^. Raw fastq files were filtered by the Quantitative Insights into Microbial Ecology software. High-quality sequences were clustered into Operational Taxonomic Units (OTUs) with similarity ≥ 97% by USEARCH UPARSE ^9^. Then, OTUs were classified into kingdom, phylum, class, order, family and genus levels according to the Greengenes database ^10^, and eventually an OTU table was created. Alpha diversity (Chao1/Shannon/Simpson) was used to reflect the diversity indices. Partial least-squares discriminant analysis (PLSDA)^11^ ^12^ and Principal Co-ordinates Analysis (PCoA) based bray curtis distance^13^ was used to reflect β diversity. Wilcoxon rank-sum test was applied to identify the differentially abundant taxa between the S and NS groups (it was considered statistically significant when *p* ≤ 0.05) ^9^.

***1.7. Non-target metabolomics analysis ^14, 15^***

Non-target metabolomics approach was applied for investigating differences in general metabolic profiles between sensitive and non-sensitive individuals.

*1.7.1. GC-MS analysis*

*Sample preparation.* About 20 mg fecal samples were homogenized with precooled saline (1:4, mg/μL). Methanol was added to the fecal homogenate (4:1, μL/μL) and vortex-mixed for 15 min to precipitate protein and extract metabolites. After two times centrifugation (4 °C, 12000 rpm, 10 min), 80 μL of the supernatant was transferred to a brown glass vial. The mixture was incubated at 37 °C for 90 min after adding with 25 μL methoxyamine hydrochloride (10 mg/mL in pyridine). Then, the mixture was vacuum dried at 50 °C for 2 h (Labconco CentriVap®, Kansas, USA). After that, 120 μL of MSTFA was added to the vial for chemical derivatization, and the mixture was incubated at 37 °C for 2 h. In the end, the supernatant was collected for GC-MS analysis.

*Instrument parameters.* Compound separation was performed on an Rtx-5MS capillary column (30.0 m × 0.25 mm × 0.25 μm), and compounds were detected by a GC-MS-QP2010 Ultra (Shimadzu Inc., Kyoto, Japan). Column temperature was initially set at 70 °C for 2 min and increased from 70 to 320 °C (10 °C/min) from 2 to 27 min, and then maintained at 320 °C for 2 min. The flow rate of carrier gas (Helium) was 1.0 mL/min. The temperatures of the injector, transfer line and ion source were maintained at 250, 250, and 200 °C, respectively. Injection volume was 1 μL with a split ratio at 30:1. Data acquisition was performed in full scan mode (from m/z 45 to 600); Mass spectrometer was operated in electron impact mode (EI, 70 eV).

*Metabolites annotation.* Metabolite features detected by GC-MS was annotated by comparing the mass spectra with those available in National Institute of Standards and Technology (NIST 11) library (similarity should be 80% or more), and further confirmed with available standard compounds in the laboratory by retention time, accurate mass as well as mass spectra.

*1.7.2. LC–MS/MS analysis*

*Sample preparation.* The procedures of sample preparation for LC-MS/MS analysis were same to those for GC-MS analysis except that there were no chemical derivatization steps, i.e., the supernatant taken from methanol extraction was transferred to a LC-MS vial for instrument analysis.

*Instrument parameters.* LC-MS/MS analysis was carried out on an UFLC system coupled with ion trap/time-off light hybrid mass spectrometry (UFLC-IT-TOF/MS, Shimadzu Inc., Kyoto, Japan), and compounds were separated by a Phenomenex Kinetex C18 column (100 × 2.1 mm, 2.6 μm). The mobile phase was consisting of (A) 0.1% formic acid in water and (B) acetonitrile. The eluting gradient was 30 min and described as follows: mobile phase A was decreased from 95 to 5%, within 20 min, maintained at 5% A for 3min, then brought back to 95% and maintained there for 7 min. Flow rate was 0.4 mL/min. Column temperature was set at 40 °C; Injection volume was 5 μL; Electrospray ionization (ESI) source in both positive and negative modes with m/z ranging from 100 to1000 was applied; TOF analyzer detector voltage was 1.80 kV. Curved desorption line (CDL) and heat block temperature were both set at 200 °C. Interface voltage was 4.5 kV for positive mode and -3.5 kV for negative mode.

*Metabolites annotation.* Compound formula was firstly predicted based on theoretical and observed m/z values as well as isotopic patterns performed by Formula Predictor in LC/MS Solution software. Then, metabolites were annotated by comparing the m/z values, formulae and the MS/MS fragmentations with to online databases, such as HMDB (<http://www.hmdb.ca>), the Mass Bank (<http://www.massbank.jp>) and METLIN Metabolite (<http://metlin.scripps.edu>), etc. Further confirmation was done by comparing with available standard compounds with respect to retention time, accurate mass as well as mass spectra.

*1.7.3. Data analysis and differential metabolites screening*

Quality control (QC) was generated by pooling equal aliquot of each sample and was processed together with actual samples. QC was injected every seven samples in the analytical sequence to check the robustness of the non-target metabolomics workflow. The variable importance in projection (VIP) generated from orthogonal partial least-squares-discriminant analysis (OPLS-DA) models and *p* values from non-parametric Mann–Whitney U test (SPSS 19.0, Chicago, USA) were used to determine whether a feature is significantly different between the two groups or not. Only features with VIP > 1 and *p* < 0.05 were considered for metabolite annotation.

***1.8. Bacterial Strains and Growth Conditions***

*Staphylococcus sp* CICC 10691 and *Sphingomonas sp* CICC 10509 was purchased from China Center of Industrial Culture Collection (CICC, Beijing, China). *Jeotgalicoccus sp* JSM 077023 was purchased from The Query Network for Microbial Species of China (Beijing, China). *Prevotella sp* DSM 29996 was purchased from German Collection of Microorganisms and Cell Cultures GmbH (DSMZ, Brunswick, Germany). The *Staphylococcus* was cultured in LB Medium (CM0033) at 28 °C. The *Jeotgalicoccus* was cultured in Agar Medium at 28 °C. The *Sphingomonas* was cultured in R2A Agar Medium (CM0904) at 28 °C. The *Prevotella* was cultured in Chopped Meat Medium at 37 °C under anaerobic conditions.

***1.9. Bacteria Transplantation***

After acclimatization, broad-spectrum antibiotics ABX (100 mg/kg Vancomycin, 200 mg/kg Neomycin sulfate, 200 mg/kg Metronidazole and 200 mg/kg Ampicillin) was intragastric (i.g.) administrated to mice every day for 5 days (day 1 - 5) to deplete gut microbiota ^16^. The mice were then gavaged with specific bacterial genus (10^8^ cfu/mouse) every other day for 7 days (day 7 - 14). Fecal samples were collected at day 7 and day 14. Bacteria levels were quantified by qPCR ^17^. Detailed experiment process was shown in Figure. S2.

***1.10. Bacterial DNA Extraction and Quantification by Quantitative Polymerase Chain Reaction (qPCR)***

Total bacterial genome DNA isolation and quantification were conducted as mentioned above. qPCR was performed by SYBR Green Ι Master (Roche Diagnostics, Basel, Switzerland) on a LightCycler 480 instrument (Roche). Primer sequences were listed in Table. S4.

***1.11. Effects of Bacteria Transplantation on FOLFOX efficacy***

The effects of aerobic bacteria (*Staphylococcus*, *Jeotgalicoccus* and *Sphingomonas*) and anaerobic bacteria (*Prevotella*) transplantation on FOLFOX efficacy was verified independently. After acclimatization, mice received ABX for 5 days (day -14 - -9). CT-26 cell suspension (about 10^6^ cells/mouse) was subcutaneous injected to develop xenograft models (day -7). Meanwhile, mice were gavaged with specific bacterial genus (10^8^ cfu/mouse, every other day) to establish bacteria colonization models, respectively (day -7 - 0). When tumor volume reached to about 100 mm^3^, mice were randomly divided into model groups and treatment groups (day 0). Meanwhile, fecal samples were collected on day 1. FOLFOX was intraperitoneal administrated on a weekly basis (day 2&9). Tumor volume was monitored by a vernier caliper throughout the experiment. All the mice were sacrificed after 12 days treatment of FOLFOX, tumors were removed and processed for immunohistochemistry analysis. Detailed experiment processes were shown in Figure. S14 (aerobic bacteria) and Figure. S15 (anaerobic bacteria).

***1.12. Target metabolomics analysis of bile acid ^18^***

*Sample preparation.* Fecal samples of Model (Combination of the samples from Model and FOLFOX groups, n=20), ABX (Combination of the samples from ABX and ABX-FOLFOX groups, n=20) and *Prevotella* (Combination of the samples from ABX-Pre and ABX-Pre-FOLFOX groups, n=20) were detected. About 30 mg fecal samples were homogenized with precooled saline (1:4, mg/μL). Methanol spiked with cortisone acetate at 5 μg/mL (internal standard) was added to the fecal homogenate (4:1, μL/μL). After vortex-mixing for 15 min, the mixture was centrifuged twice (4 °C, 12000 rpm, 10 min), and then the supernatant was collected for LC-MS/MS analysis.

*Instrument parameters.* The quantification of bile acids were performed on a triple quadruple TSQ Quantum mass spectrometer with ESI interface (ThermoFisher, Palo Alto, CA, USA) using a ZORBAX Eclipse XDB-C18 column (2.1mm×150 mm, 3.5 μm, Agilent, CA, USA). The mobile phase consisted of (A) acetonitrile and (B) 0.1% formic acid in water. Gradient elution with the flow rate of 0.45 mL/min was 0-20min 25% A, 20-65min 25%-40% A, and 65-70 min 25% A; Column temperature was set at 45 °C. Injection volume was 5 μL. The electrospray ionization source was performed in negative mode with spray voltage 3.8 kV and capillary temperature of 380 °C. Scan width for multiple-reaction monitoring (MRM) was 0.1 m/z.

Optimum MS parameters of each analyte for bile acids analysis were shown in Table. S5. Representative chromatograms of bile acids were shown in Figure. S16.

***1.13. 3a-HSDH sequence collection and taxonomic characterization***

Protein sequences of 3α-HSDH in bacteria were obtained from NCBI database by querying ‘3-alpha-hydroxysteroid dehydrogenase’. The total number of amino acids in the sequences was limited to 0.5 - 1.5 folds of the average sequence length. Then, the 3α-HSDH sequences were identified through HMP database and BLASTP with sequence identity of 45% as cutoff. The metagenomics sequence data of individuals collected from 11 populations was based on our previous research ^19^. Bacterial genera and related species with relative abundance ranks in top 20 were included for sequence comparison. Primer sequences for *Prevotella buccae* and 3α-HSDH were listed in Table. S3.

***1.14. Cell Proliferation Assays***

The cell proliferation assays were performed by MTT and Colony formation assay. CT-26 cells were seeded into 96-well plates in 200 μL of medium per well. After 24 h, the cells were treated with 3-Oxo at 20, 50, 100, 200 μM for additional 72 h. The cells were treated with 5 mg/mL of MTT solution (20 μL/well) for 4 h at 37 °C. After that, the medium was discarded and DMSO (150 μL/well) was added, and then the absorbance was measured at 490 nm with a microplate reader (Tecan, Mannedorf, Switzerland). Colony formation assay were performed in 6-well plates as previously described ^3^. The results are presented as mean ± standard deviation (SD). For all the assays, triplicates were performed in parallel.

***1.15. Migration assay***

CT-26 were seeded into 6-well plates and incubated in complete medium to 90% confluence. A sterilized pipette tip was used to generate wounding across the cell monolayer. Then, the cells were washed twice with PBS, added with fresh media and treated with 3-Oxo at 20, 50, 100, 200 μM for another 18 h. The cells migrating into the wounded area were visualized and photographed under the inverted microscope (Nikon TS100, Tokyo, Japan). Transwell chambers were performed as previously described ^3^.

***1.16. Western blot***

The total cell lysates were extracted with lysis buffer containing protease and phosphatase inhibitors. The proteins were fractionated by 6–15% sodium dodecyl sulfate polyacrylamide gel electrophoresis (SDS-PAGE) and then transferred to PVDF membrane (Millipore, United States). The membranes were blocked with 5% BSA-PBST for 1.5 h at room temperature and then incubated with primary antibodies (diluted in 5% BSA-PBST) for 12-14 h at 4 ℃. Next, they were probed with secondary antibodies for 1.5 h at room temperature. The expression of the target proteins was detected by the Immobilon Western chemiluminescent HRP Substrate (Millipore, United States).

***1.17. Enzyme-linked immunosorbent assay (ELISA)***

The secreted level of IL-1β, IL-8 and Tumor Necrosis Factor-α (TNF-α) in culture medium was measured using ELISA kits (4A Biotech Co.,Ltd., Beijing, China) according to the manufacturer’s instructions.

***1.18. Statistical analysis***

Spearman’s correlation analysis was applied to test the correlation between fecal bacteria levels and fecal metabolite intensities (SPSS 19.0, Chicago, USA). Data analysis and graphing were performed by GraphPad Prism 8 software (GraphPad Software Inc., La Jolla, CA, USA). The results were presented as mean ± SD, independent unpaired two-tailed Student’s t test was performed to evaluate the differences between two groups, unless otherwise specified.

**2. Supplementary Figures**

**
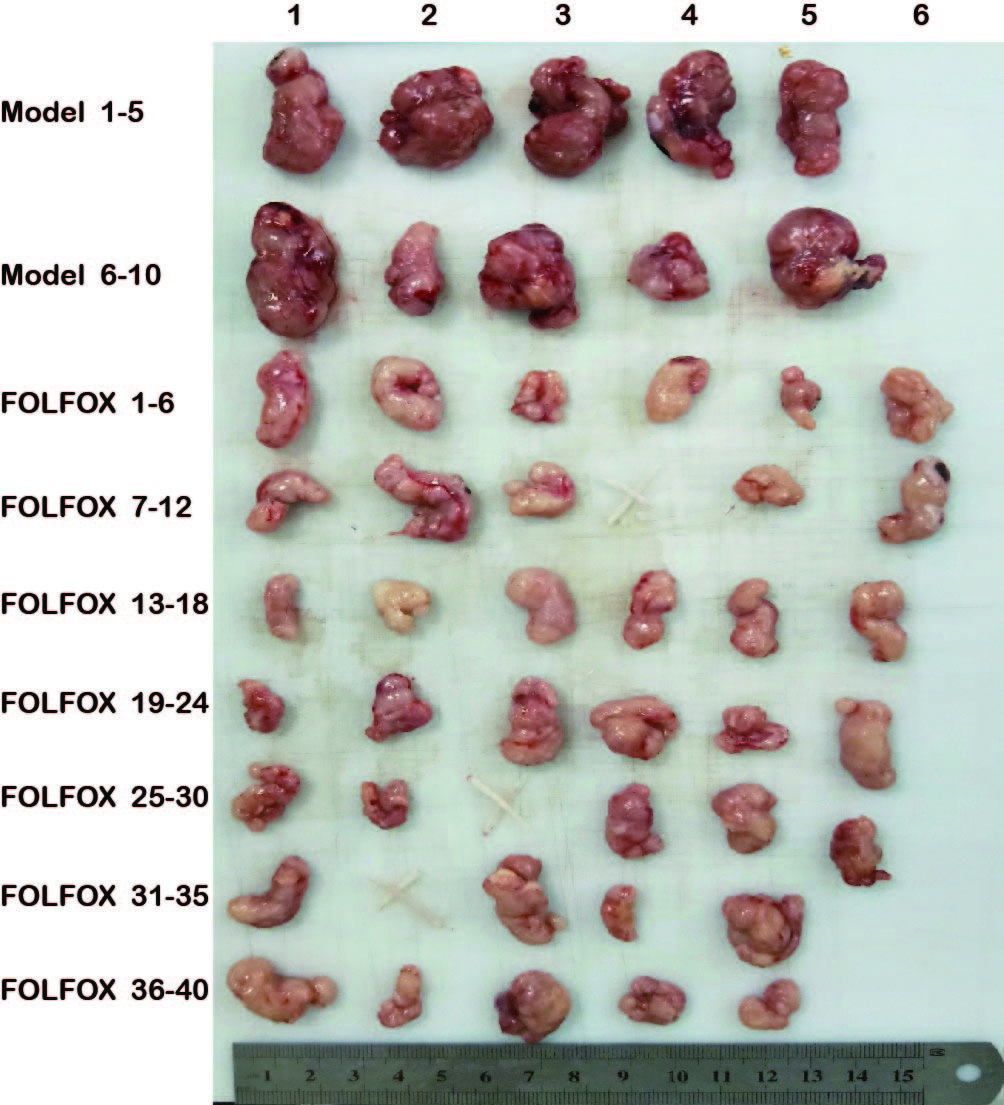
**

**Figure S1** Photo of tumors collected from Model and FOLFOX mice.


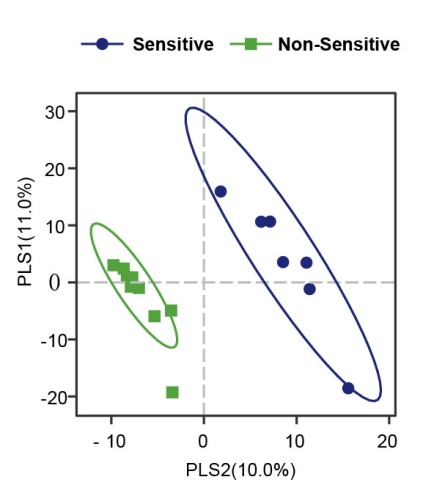


**Figure S2** Partial least-squares discriminant analysis (PLS-DA) score plot of β-diversity analysis between S and NS groups.


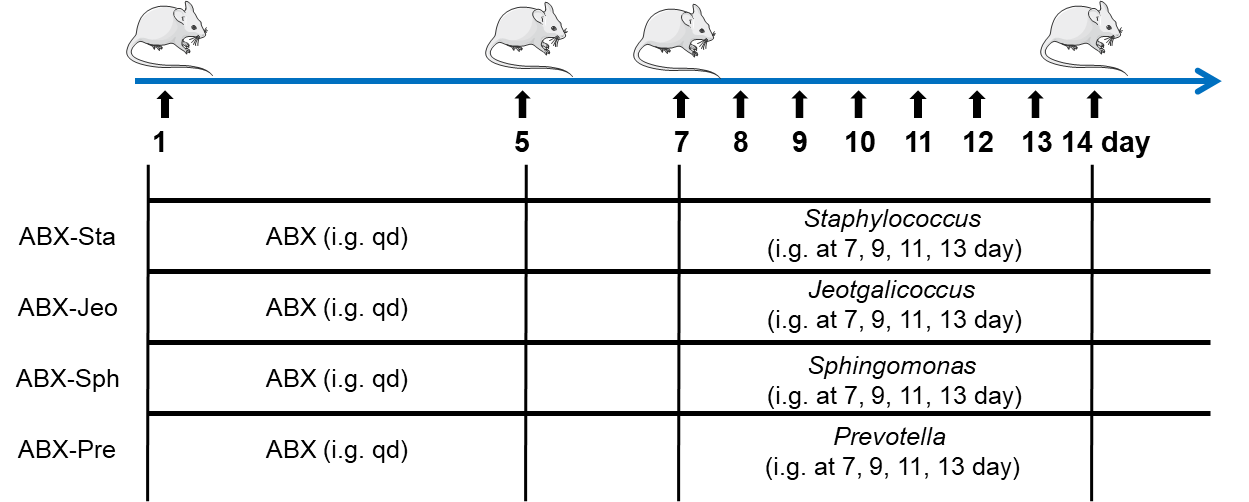


**Figure S3** Schematic of the bacteria transplantation experiment. ABX: 100 mg/kg Vancomycin, 200 mg/kg Neomycin sulfate, 200 mg/kg Metronidazole, 200 mg/kg Ampicillin; qd: once a day.

**
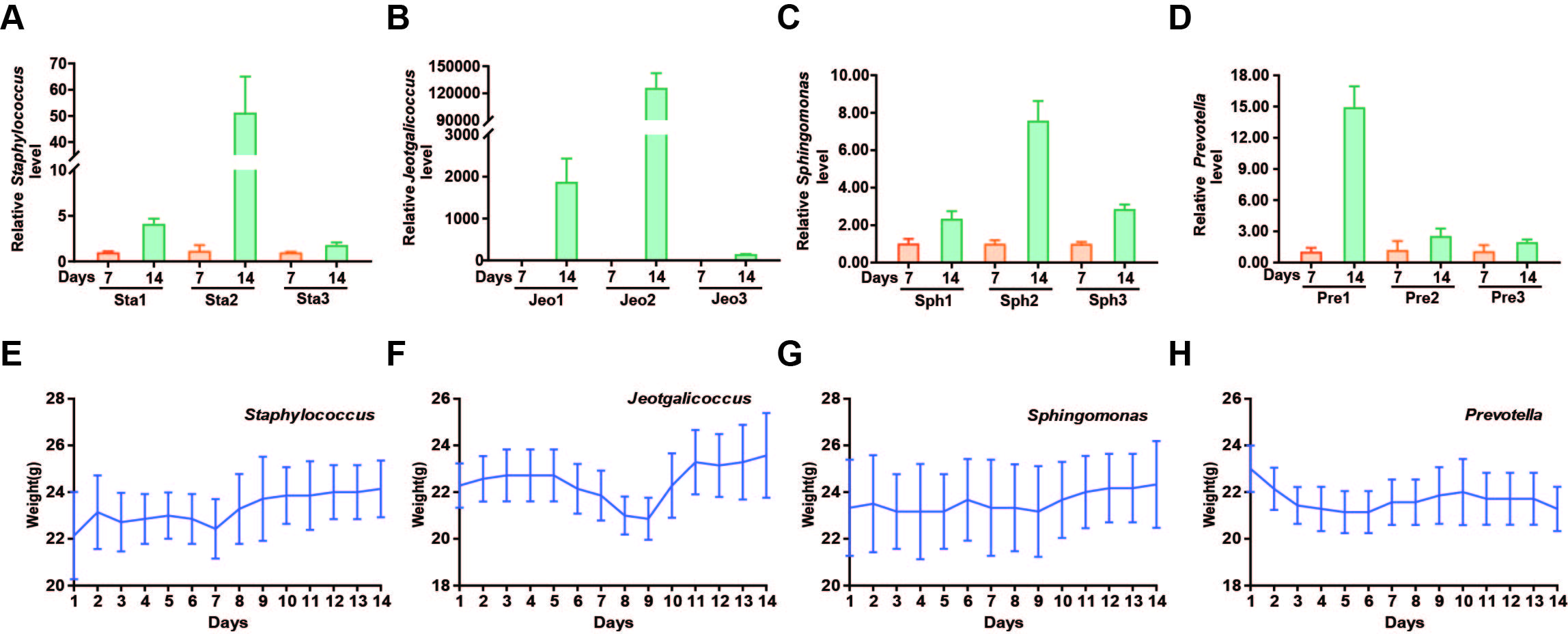
**

**Figure S4** Relative bacteria levels after transplantation. The relative level of specific bacterial genus before the transplantation was defined as 1.00 (day 7). (A-D) Relative bacteria levels before (day 7) and after (day 14) the transplantation. (E-H) Body weight of mice during experiment. Three mice were randomly selected in each group for qPCR analysis (labeled with i.e., Sta1, Sta2 and Sta3) and qPCR assays were repeated for three times.


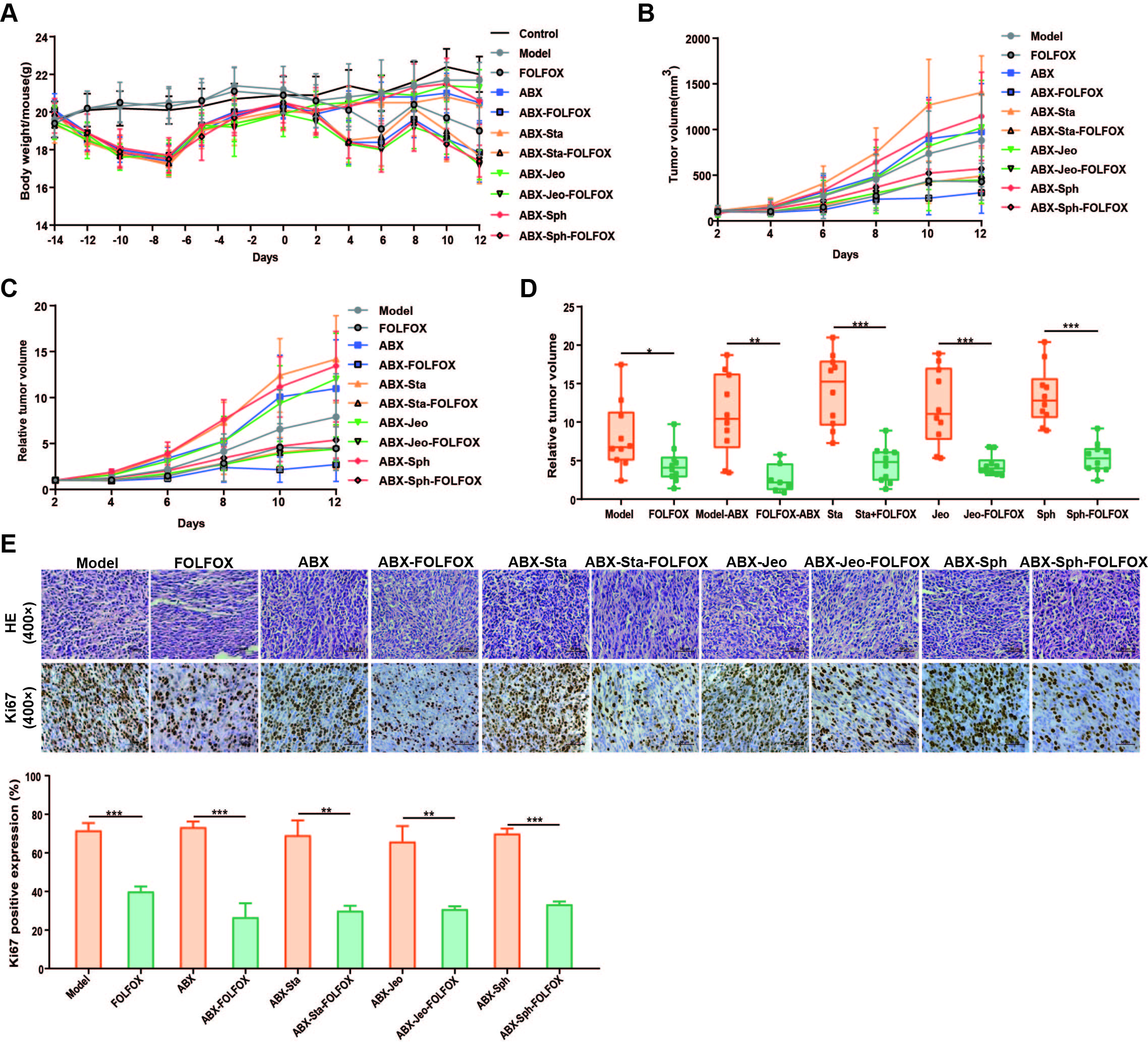


**Figure S5** Effects of aerobic bacteria transplantation on FOLFOX efficacy. (A) Change of mice body weight across the experiment. Tumor volume (B) and relative tumor volume (C, D) were measured throughout the experiment. Inhibition rate: Model *vs.* FOLFOX (45.25%); ABX *vs.* ABX-FOLFOX (75.39%); ABX-Sta *vs.* ABX-Sta-FOLFOX (67.66%); ABX-Jeo *vs.* ABX-Jeo-FOLFOX (63.56%); ABX-Sph *vs.* ABX-Sph-FOLFOX (59.95%). (E) Ki67 levels were compared in Model *vs.* FOLFOX group, ABX *vs.* ABX-FOLFOX group, ABX-Sta *vs.* ABX-Sta-FOLFOX group, ABX-Jeo *vs.* ABX-Jeo-FOLFOX group, and ABX-Sph *vs.* ABX-Sph-FOLFOX group. Data was expressed as mean ± SD. It was considered statistically significant when *p* < 0.05, **p*<0.05, ***p*<0.01, ****p*<0.001.

**
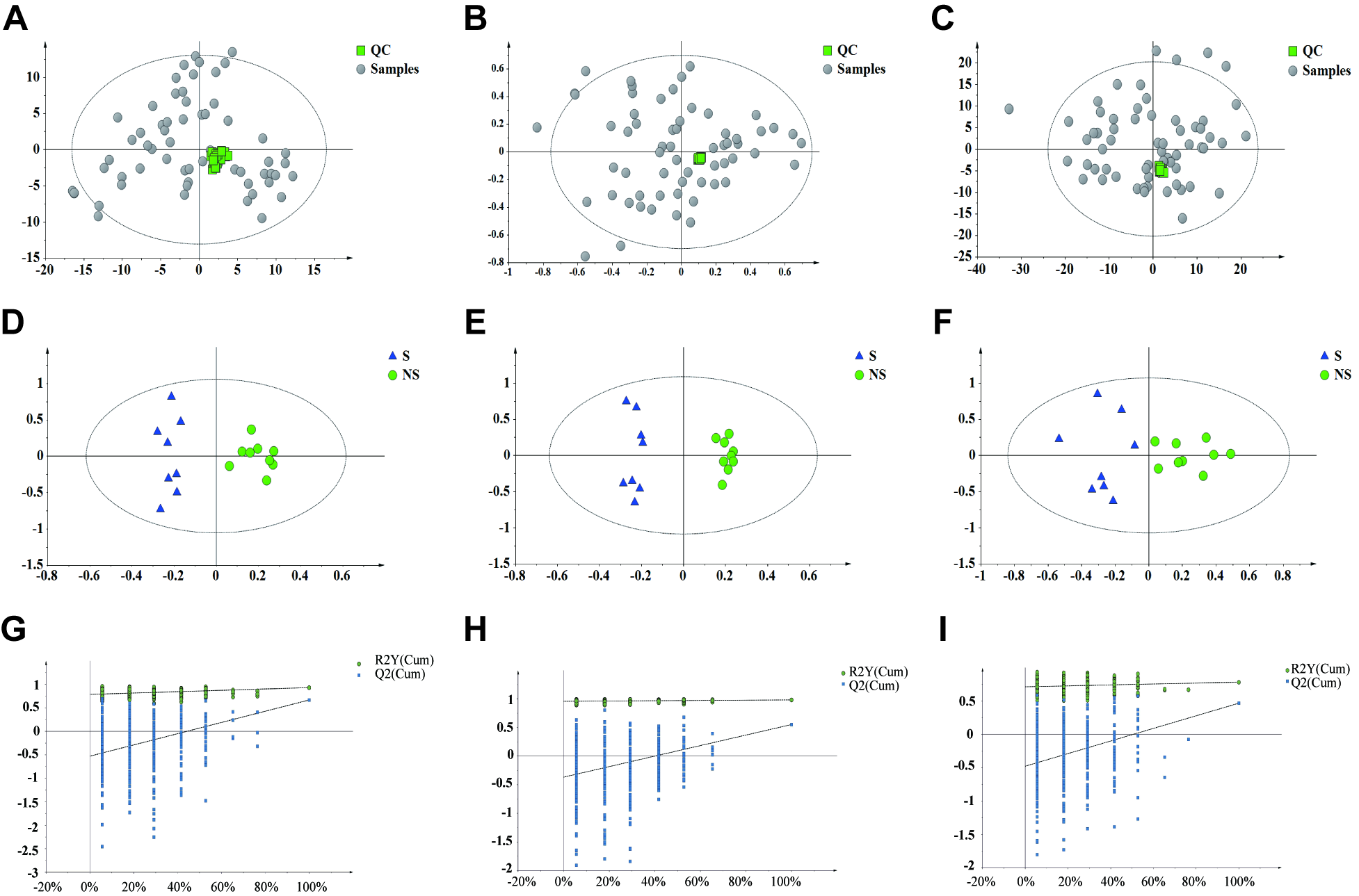
**

**Figure S6** Non-target metabolomics analysis of pre-dose fecal samples from S and NS mice. (A-C) QC samples were clustered very well in PCA score plots derived from GC-MS, LC-MS (+) and LC-MS (-) datasets. (D-F) OPLS-DA score plot based on GC-MS (R^2^X=0.519, R^2^Y=0.932, Q^2^=0.667), LC-MS (+) (R^2^X=0.617, R^2^Y=0.984, Q^2^=0.553) and LC-MS (-) (R^2^X=0.449, R^2^Y=0.782, Q^2^=0.466) data, respectively. (G-I) Permutation test result (500 times) of OPLS-DA models constructed from GC-MS, LC-MS (+) and LC-MS (-) data, respectively.


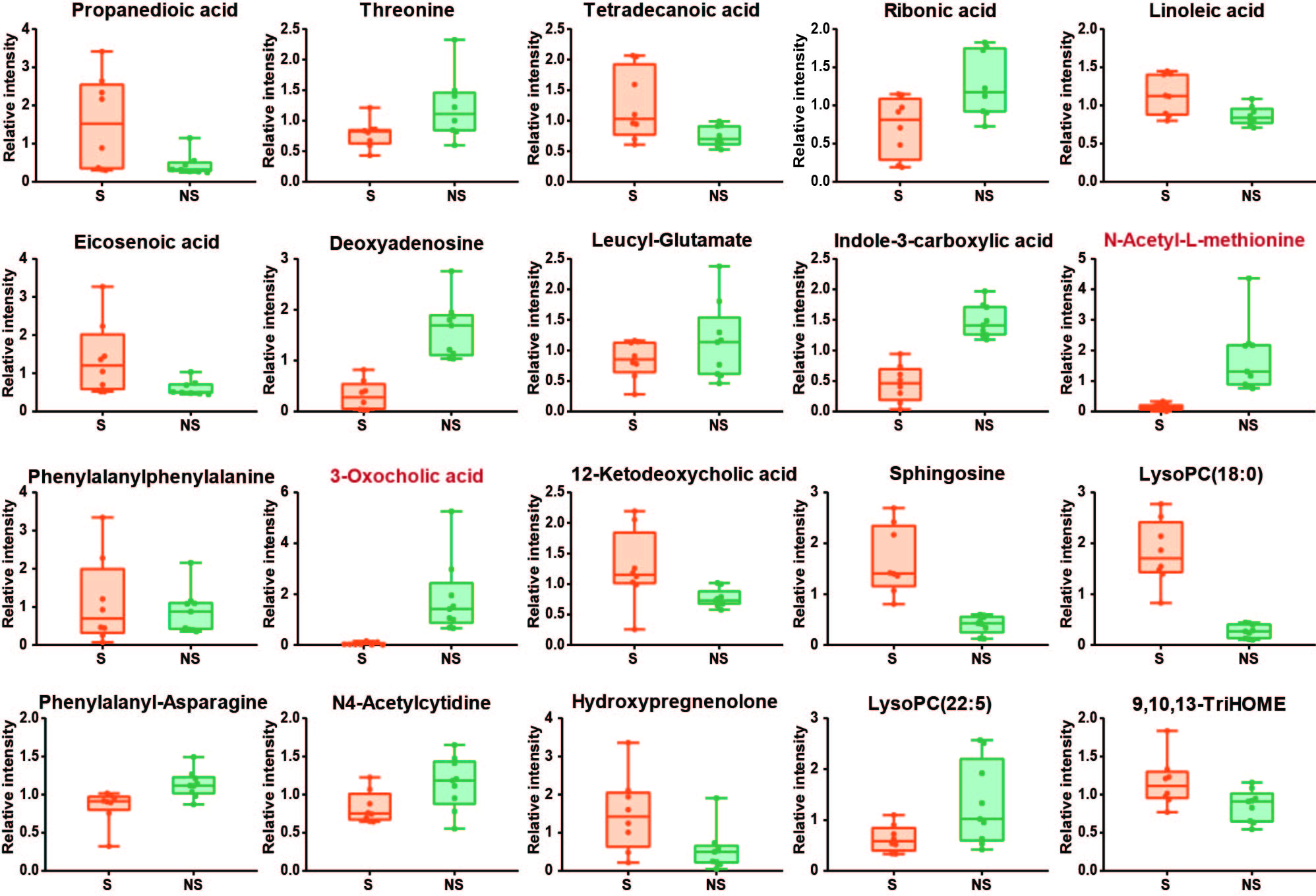


**Figure S7** Relative abundance of twenty differential metabolites in pre-dose fecal samples between the S (n=8) and NS (n=9) group.

**
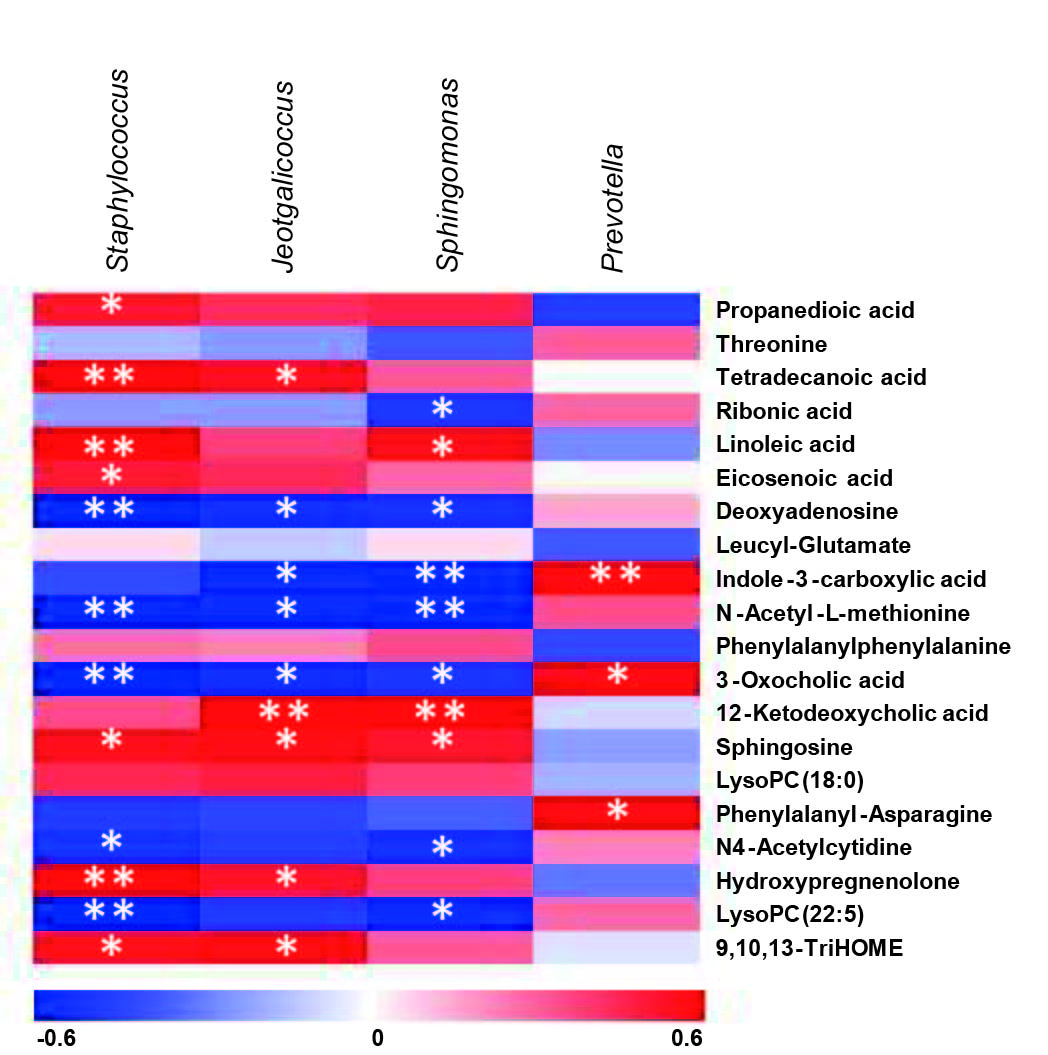
**

**Figure S8** Heatmap of Spearman correlation coefficient between the changed twenty fecal metabolites and four bacterial genera. The intensity of the colors represents the degree of association between the level of fecal metabolites and bacterial genera abundance measured by Spearman’s correlations. It was considered statistically significant when *p* < 0.05, **p*<0.05, ***p*<0.01.


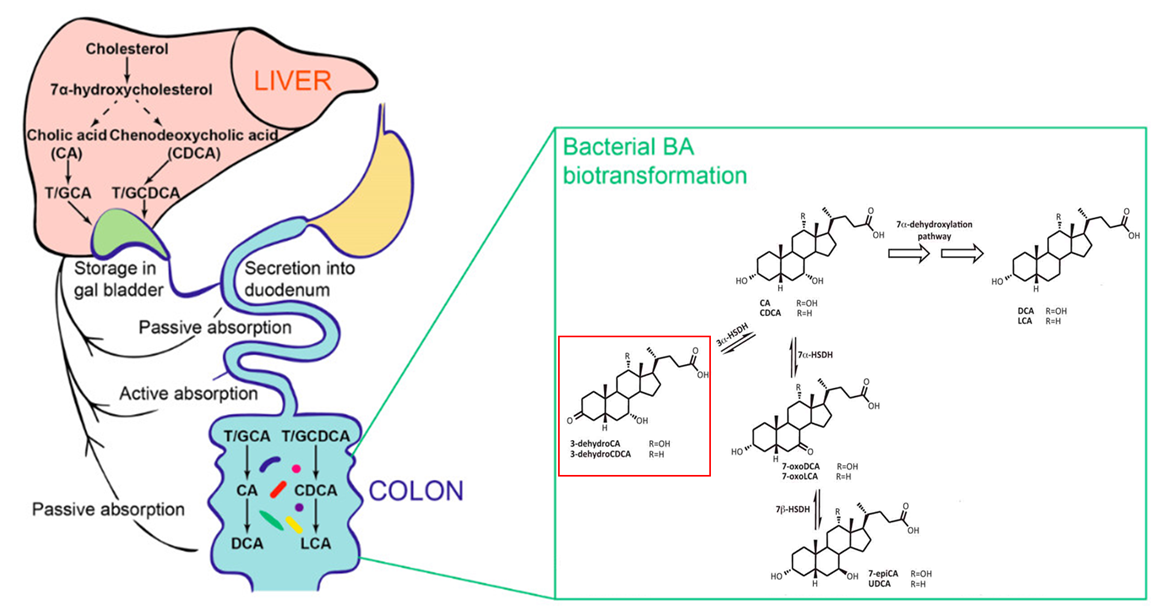


**Figure S9** Bile acid biotransformation pathway mediated by intestinal microbiota ^20, 21^.


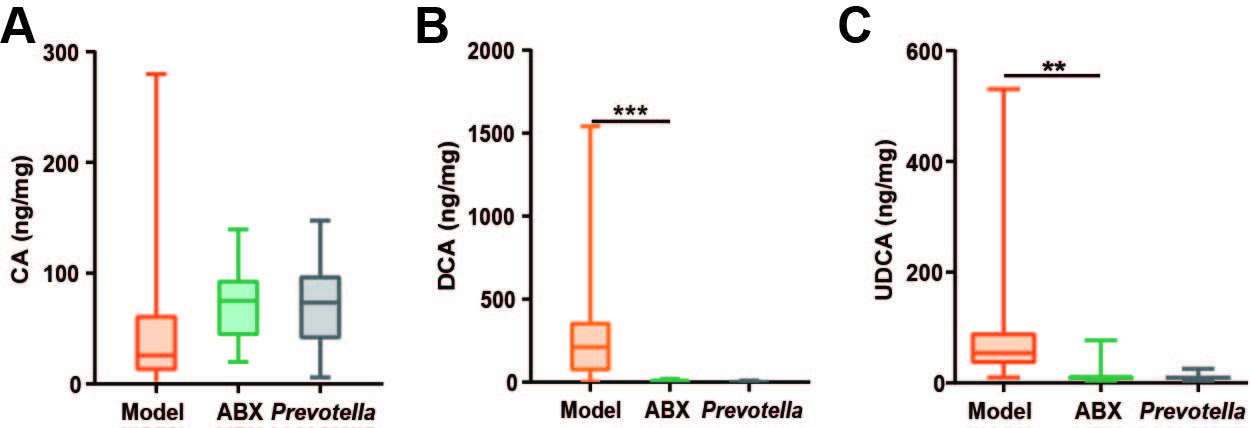


**Figure S10** Concentrations of bile acids including (A) CA, (B) DCA, and (C) UDCA in fecal samples from the Model, ABX and *Prevotella* group. CA-Cholic acid; DCA-Deoxycholic acid; UDCA-Ursodeoxycholic acid.

**
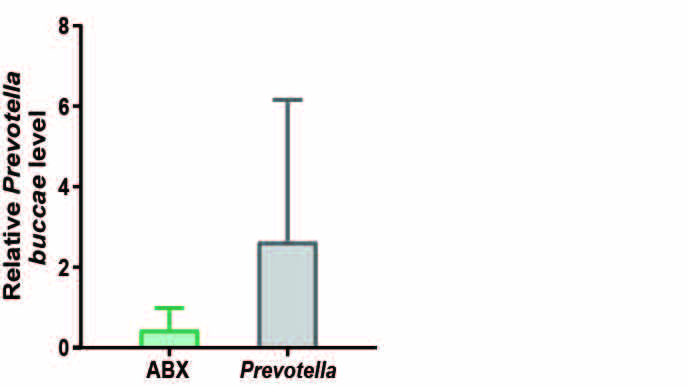
**

**Figure S11** Relative levels of *Prevotella buccae* in fecal samples detected from the ABX and *Prevotella* groups.

**
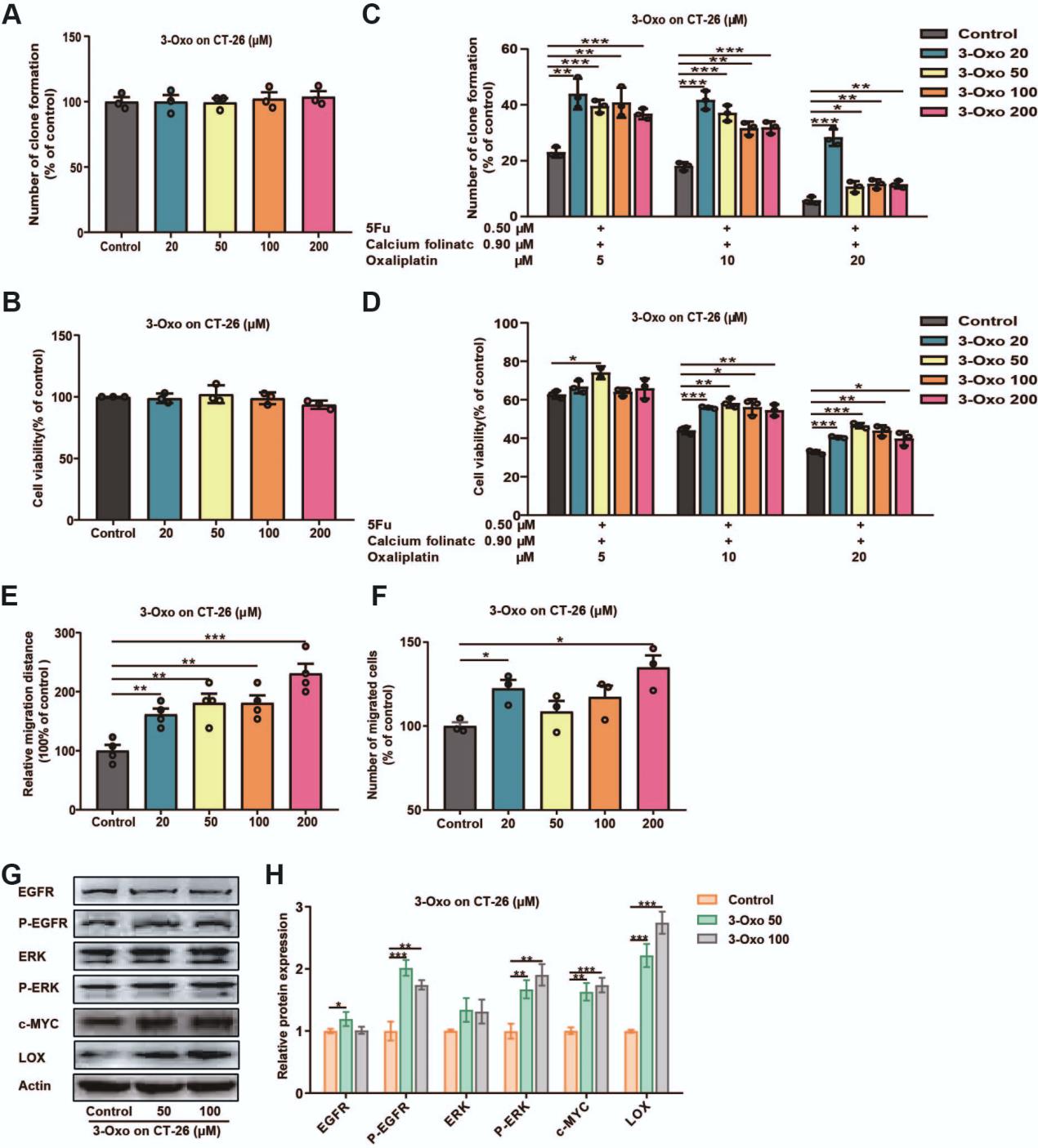
**

**Figure S12** Effects of 3-Oxo on cancer development as well as on the anti-cancer effect of FOLFOX. (A-B) The effect of 3-Oxo on CT-26 proliferation was evaluated by MTT (72h) and colon formation assay (7days). (C-D) 3-Oxo could attenuate the anti-proliferation effect of FOLFOX (72h, 7days). (E-F) The effect of 3-Oxo on CT-26 migration was evaluated by wound-healing assay and transwell chambers. (G) 3-Oxo upregulated the expression of EGFR/ERK/c-MYC and LOX in CT-26 (72h). (H) Quantification of the band intensity of (G). Data were expressed as mean ± SD of three independent experiments. It was considered statistically significant when *p* < 0.05, **p*<0.05, ***p*<0.01, ****p*<0.001.


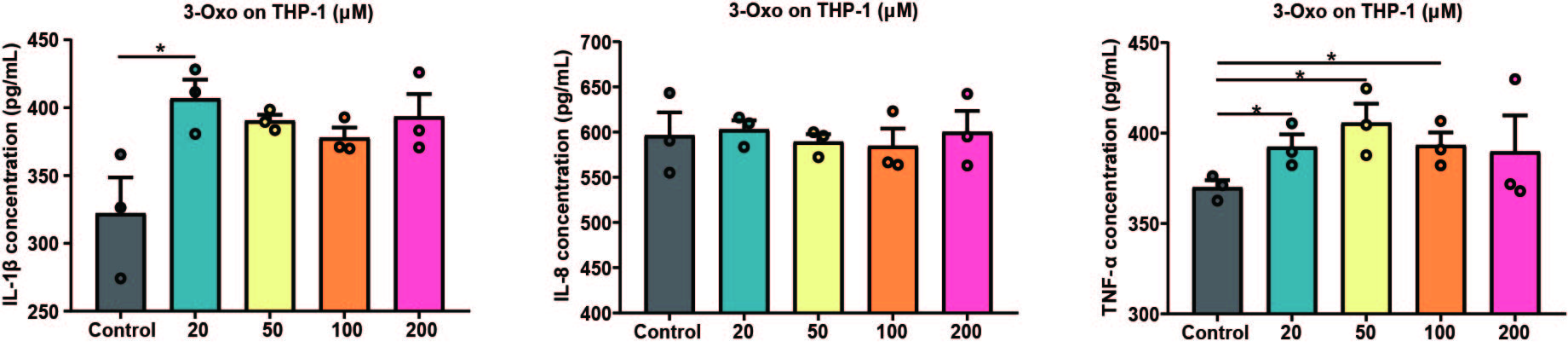


**Figure S13** 3-Oxo could induce the secretion of IL-1β and TNF-α in macrophages. Data was expressed as mean ± SD of three independent experiments. It was considered statistically significant when *p* < 0.05, **p*<0.05, ***p*<0.01, ****p*<0.001.


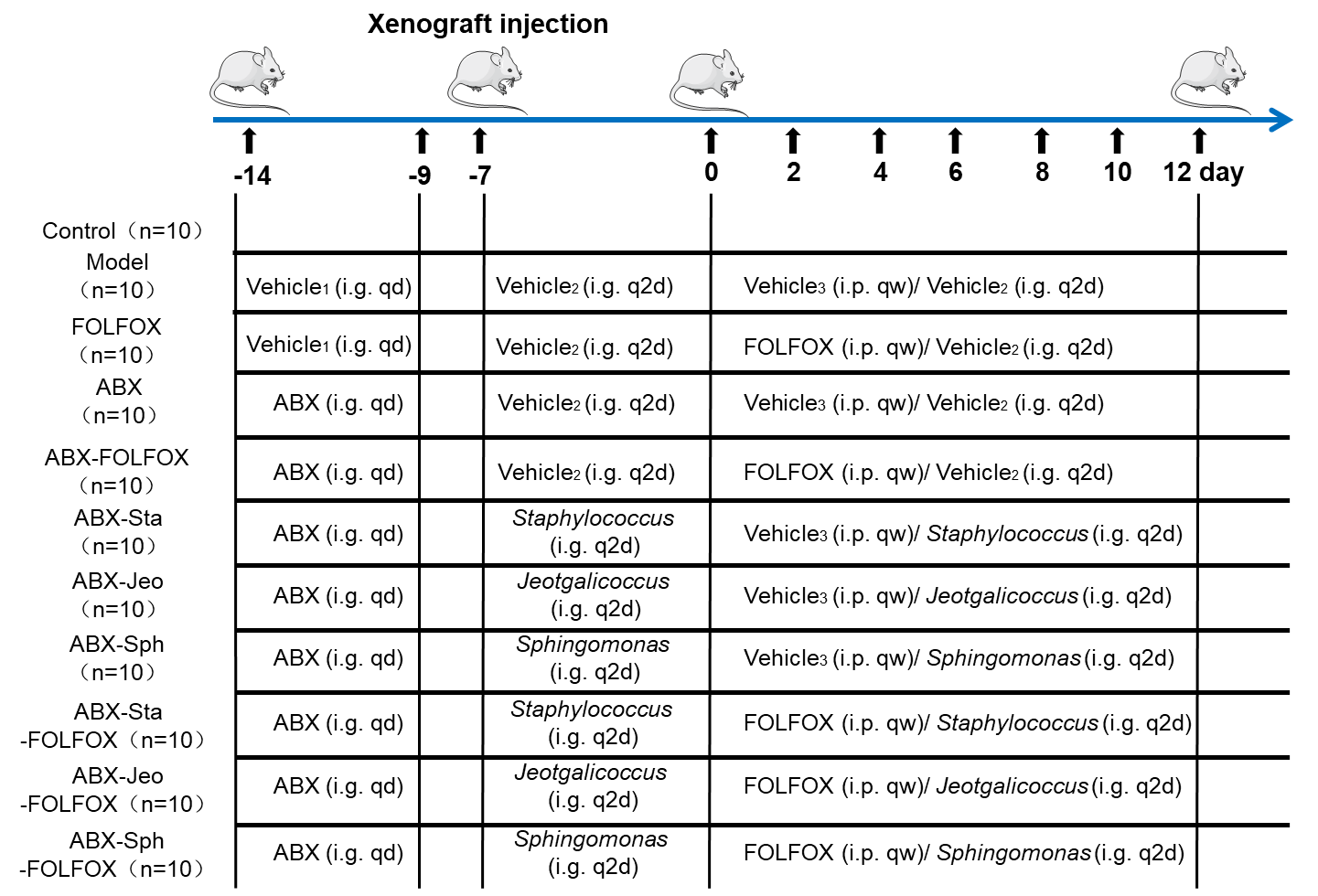
**Figure S14** Workflow of the experiment investigating aerobic bacteria transplantation on FOLFOX efficacy. FOLFOX (6 mg/kg Oxaliplatin followed by 50 mg/kg 5-Fu and 90 mg/kg Calcium Folinate in 2 hours); Vehicle_1_: Vehicles of ABX; Vehicle_2_: Vehicles of bacterial genus; Vehicle_3_: Vehicles of FOLFOX; qd-once a day; q2d-every other day; qw-once a week.


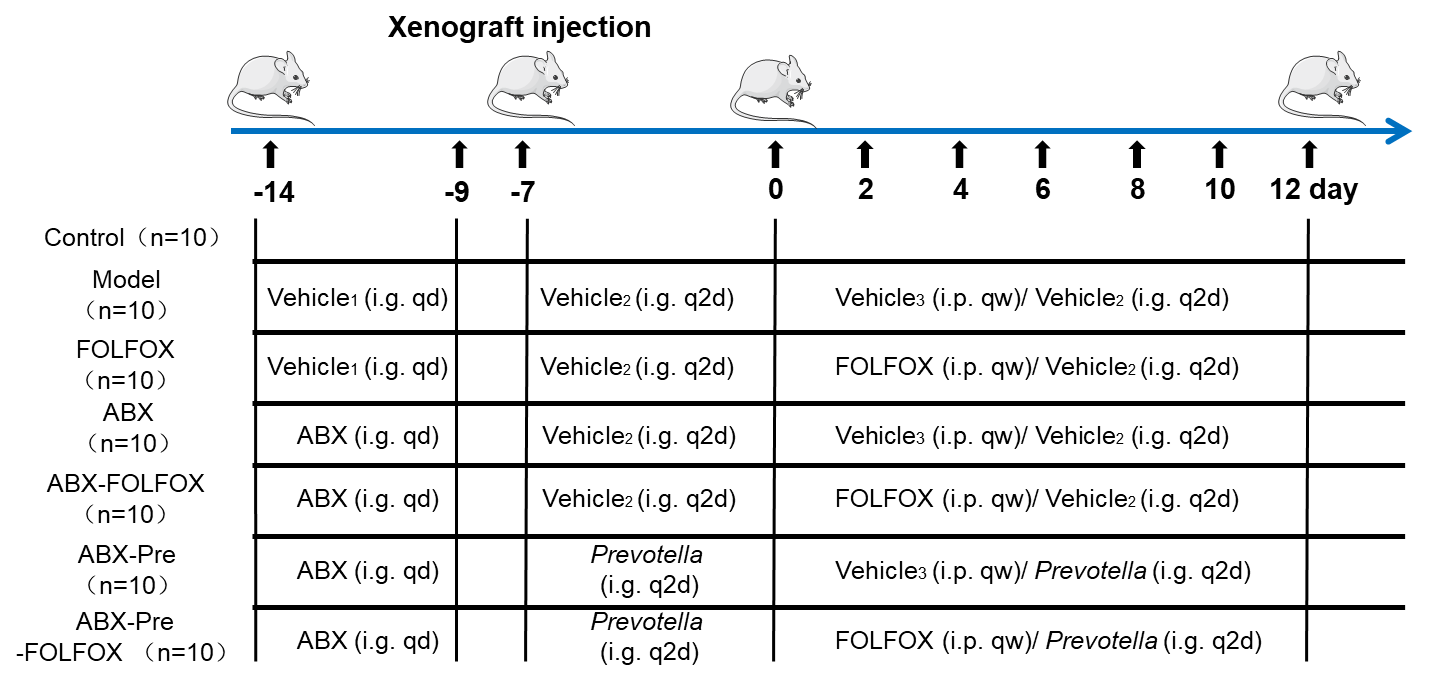


**Figure S15** Workflow of the experiment investigating anaerobic bacteria transplantation on FOLFOX efficacy.

**
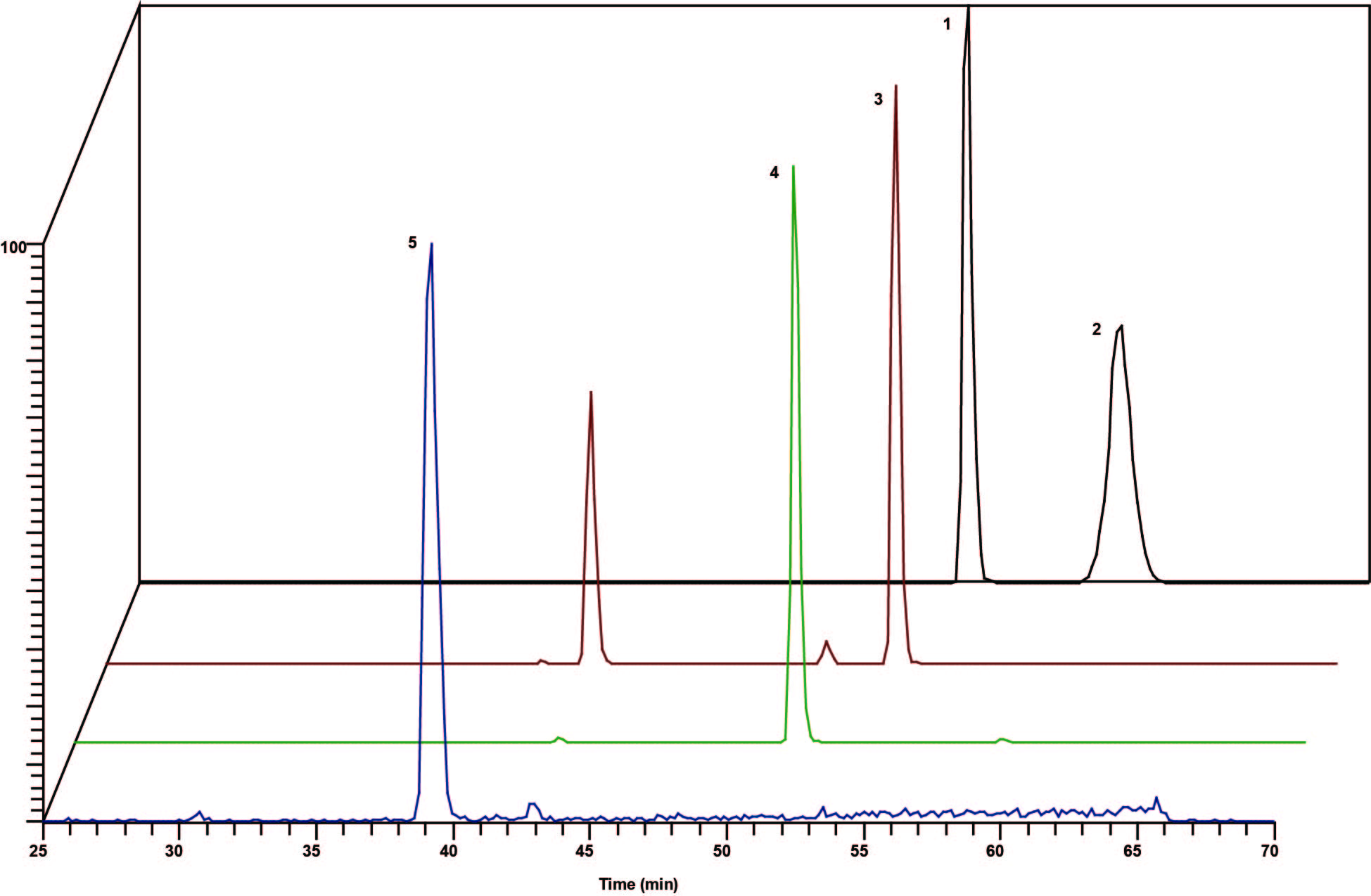
**

**Figure S16** Representative chromatograms of bile acids in target metabolomics analysis (1 – UDCA; 2 – DCA; 3 – CA; 4 – 3-Oxo; 5 – IS).

**3. Supplementary Tables**

**Table S1.** Detailed grouping scheme (Mice labeled with F10, F27 and F32 died before the end of the experiment).

| **Mice no.** | **Grouping by RTV** | **Grouping by Ki67** | **Final grouping (S or NS)** |
| --- | --- | --- | --- |
| F1 |  |  |  |
| F2 |  | S2 |  |
| F3 | S1 |  |  |
| F4 | S1 | S2 | S |
| F5 | S1 | S2 | S |
| F6 | S1 | S2 | S |
| F7 | S1 | S2 | S |
| F8 | NS1 |  |  |
| F9 |  | NS2 |  |
| ~~F10~~ |  |  |  |
| F11 | S1 | S2 | S |
| F12 |  |  |  |
| F13 |  |  |  |
| F14 |  |  |  |
| F15 | NS1 | NS2 | NS |
| F16 | NS1 | NS2 | NS |
| F17 | NS1 | NS2 | NS |
| F18 |  | NS2 |  |
| F19 | S1 | S2 | S |
| F20 | NS1 |  |  |
| F21 | NS1 | NS2 | NS |
| F22 |  | NS2 |  |
| F23 | S1 | S2 | S |
| F24 | NS1 | NS2 | NS |
| F25 |  |  |  |
| F26 | S1 | S2 | S |
| ~~F27~~ |  |  |  |
| F28 | NS1 | NS2 | NS |
| F29 | NS1 | NS2 | NS |
| F30 |  |  |  |
| F31 |  | S2 |  |
| ~~F32~~ |  |  |  |
| F33 | NS1 | NS2 | NS |
| F34 | S1 |  |  |
| F35 |  |  |  |
| F36 | NS1 | NS2 | NS |
| F37 | S1 |  |  |
| F38 | NS1 | S2 |  |
| F39 |  |  |  |
| F40 | S1 |  |  |

**Table S2.** Differential metabolites in pre-dose fecal samples between the S and NS groups.

| **No.** | Metabolites | VIP | *p* value | Fold change(S/NS) |
| --- | --- | --- | --- | --- |
| 1 | Propanedioic acid | 2.09 | 0.027 | ↑3.48 |
| 2 | Threonine | 1.09 | 0.043 | ↓1.55 |
| 3 | Tetradecanoic acid | 3.03 | 0.034 | ↑1.68 |
| 4 | Ribonic acid | 1.38 | 0.027 | ↓1.77 |
| 5 | Linoleic acid | 1.22 | 0.034 | ↑1.31 |
| 6 | Eicosenoic acid | 1.41 | 0.021 | ↑2.28 |
| 7 | Deoxyadenosine | 2.52 | 0.004 | ↓5.18 |
| 8 | Leucyl-glutamate | 1.36 | 0.004 | ↓1.34 |
| 9 | Indole-3-carboxylic acid | 1.69 | 0.001 | ↓3.21 |
| 10 | N-acetyl-L-methionine | 1.82 | 0.007 | ↓12.56 |
| 11 | Phenylalanylphenylalanine | 1.78 | 0.034 | ↑1.27 |
| 12 | 3-Oxocholic acid | 1.24 | 0.009 | ↓33.83 |
| 13 | 12-Ketodeoxycholic acid | 2.06 | 0.034 | ↑1.64 |
| 14 | Sphingosine | 3.09 | 0.007 | ↑4.10 |
| 15 | LysoPC(18:0) | 3.01 | 0.021 | ↑6.71 |
| 16 | Phenylalanyl-asparagine | 1.44 | 0.004 | ↓1.35 |
| 17 | N4-Acetylcytidine | 1.82 | 0.043 | ↓1.38 |
| 18 | Hydroxypregnenolone | 3.25 | 0.016 | ↑2.69 |
| 19 | LysoPC (22:5) | 1.58 | 0.012 | ↑2.08 |
| 20 | 9,10,13-TriHOME | 1.04 | 0.027 | ↓1.36 |

**Table S3.** Bacterial species containing 3α-HSDH paralogs.

| Species | Blast results with cutoff 45% sequence identity |
| --- | --- |
|  | 3α-HSDH |
| *Akkermansia muciniphila* | 1/1 |
| *Bacteroides coprocola* | 1/1 |
| *Bacteroides dorei* | 2/2 |
| *Bacteroides fragilis* *638R* | 1/3 |
| *Bacteroides plebeius* | 1/1 |
| *Bacteroides sp. 3_1_33FAA* | 1/1 |
| *Bacteroides sp. 3_2_5* | 1/1 |
| *Bacteroides sp. 9_1_42FAA* | 1/1 |
| *Clostridium butyricum* | 1/1 |
| *Eubacterium eligens* | 1/1 |
| ***Prevotella buccae*** | **1/1** |
| *Ruminococcus albus* | 1/1 |
| *Ruminococcus flavefaciens* | 1/1 |

**Table S4.** PCR primers.

| Primer | Direction | Sequence (5’ → 3’) |
| --- | --- | --- |
| Total bacteria detection | Forward | GTGSTGCAYGGYTGTCGTCA |
|  | Reverse | ACGTCRTCCMCACCTTCCTC |
| *Staphylococcus* | Forward | GCGTGGGGATCAAACAGGAT |
|  | Reverse | GTTCTTCGCGTTGCTTCGAA |
| *Jeotgalicoccus* | Forward | TCACCAAGGCGACGATGCA |
|  | Reverse | GCCAGTTACTACCTCAAGTGTTCT |
| *Sphingomonas* | Forward | GCGACGATCCTTAGCTGGTC |
|  | Reverse | GCATTGCTGGATCAGGCTTT |
| *Prevotella* | Forward | CCAGCCAAGTAGCGTGCA |
|  | Reverse | TGGACCTTCCGTATTACCGC |
| *Prevotella buccae* | Forward | CGGTAGGGGTTCTGAGAGGA |
|  | Reverse | GTTTACCGTGCGGACTACCA |
| 3α-HSDH | Forward | ATTAGTAACGGGAGCCGCAG |
|  | Reverse | TGCCAATCACTTCATCGCCT |

**Table S5.** Optimized MS parameters.

| Analyte | Precursor ion (*m/z*) | ESI mode | Collision energy (V) | Product ion (*m/z*) |
| --- | --- | --- | --- | --- |
| CA | 407.2 | - | 20 | 407.2 |
| DCA | 391.2 | - | 20 | 391.2 |
| 3-Oxo | 405.2 | - | 20 | 405.2 |
| UDCA | 391.2 | - | 20 | 391.2 |
| IS | 401.20 | - | 40 | 340.50 |

**References**

1. Limani P, Linecker M, Kachaylo E, et al. Antihypoxic Potentiation of Standard Therapy for Experimental Colorectal Liver Metastasis through Myo-Inositol Trispyrophosphate. *Clinical cancer research : an official journal of the American Association for Cancer Research*. Dec 1 2016;22(23):5887-5897. doi:10.1158/1078-0432.CCR-15-3112

2. Robinson SM, Mann J, Vasilaki A, et al. Pathogenesis of FOLFOX induced sinusoidal obstruction syndrome in a murine chemotherapy model. *J Hepatol*. Aug 2013;59(2):318-26. doi:10.1016/j.jhep.2013.04.014

3. Du H, Chen Y, Hou X, et al. PLOD2 regulated by transcription factor FOXA1 promotes metastasis in NSCLC. *Cell death & disease*. Oct 26 2017;8(10):e3143. doi:10.1038/cddis.2017.553

4. Hou X, Du H, Quan X, et al. Silibinin Inhibits NSCLC Metastasis by Targeting the EGFR/LOX Pathway. *Front Pharmacol*. 2018;9:21. doi:10.3389/fphar.2018.00021

5. Luo FR, Yang Z, Dong H, et al. Prediction of active drug plasma concentrations achieved in cancer patients by pharmacodynamic biomarkers identified from the geo human colon carcinoma xenograft model. *Clinical cancer research : an official journal of the American Association for Cancer Research*. Aug 1 2005;11(15):5558-65. doi:10.1158/1078-0432.CCR-05-0368

6. von Minckwitz G, Schmitt WD, Loibl S, et al. Ki67 measured after neoadjuvant chemotherapy for primary breast cancer. *Clinical cancer research : an official journal of the American Association for Cancer Research*. Aug 15 2013;19(16):4521-31. doi:10.1158/1078-0432.CCR-12-3628

7. Guo M, Jiang W, Luo J, Yang M, Pang X. Analysis of the Fungal Community in Ziziphi Spinosae Semen through High-Throughput Sequencing. *Toxins (Basel)*. Nov 25 2018;10(12)doi:10.3390/toxins10120494

8. Zhu C, Yuan C, Ao S, et al. The Predictive Potentiality of Salivary Microbiome for the Recurrence of Early Childhood Caries. *Front Cell Infect Microbiol*. 2018;8:423. doi:10.3389/fcimb.2018.00423

9. Feng YL, Cao G, Chen DQ, et al. Microbiome-metabolomics reveals gut microbiota associated with glycine-conjugated metabolites and polyamine metabolism in chronic kidney disease. *Cell Mol Life Sci*. Dec 2019;76(24):4961-4978. doi:10.1007/s00018-019-03155-9

10. Suez J, Zmora N, Zilberman-Schapira G, et al. Post-Antibiotic Gut Mucosal Microbiome Reconstitution Is Impaired by Probiotics and Improved by Autologous FMT. *Cell*. Sep 6 2018;174(6):1406-1423 e16. doi:10.1016/j.cell.2018.08.047

11. Rodrigues C, Sousa C, Lopes JA, Novais A, Peixe L. A Front Line on Klebsiella pneumoniae Capsular Polysaccharide Knowledge: Fourier Transform Infrared Spectroscopy as an Accurate and Fast Typing Tool. *mSystems*. Mar 24 2020;5(2)doi:10.1128/mSystems.00386-19

12. Wang Y, Zhang J, Zhou Q, et al. Analysis of the Intestinal Flora in Male Versus Female Swamp Eels (Monopterus albus). *Front Microbiol*. 2020;11:699. doi:10.3389/fmicb.2020.00699

13. Wu Y, Bible PW, Long S, et al. Metagenomic analysis reveals gestational diabetes mellitus-related microbial regulators of glucose tolerance. *Acta Diabetol*. May 2020;57(5):569-581. doi:10.1007/s00592-019-01434-2

14. Gao Y, Li W, Chen J, et al. Pharmacometabolomic prediction of individual differences of gastrointestinal toxicity complicating myelosuppression in rats induced by irinotecan. *Acta Pharm Sin B*. Jan 2019;9(1):157-166. doi:10.1016/j.apsb.2018.09.006

15. Zhang P, Chen JQ, Huang WQ, et al. Renal Medulla is More Sensitive to Cisplatin than Cortex Revealed by Untargeted Mass Spectrometry-Based Metabolomics in Rats. *Sci Rep*. Mar 16 2017;7:44804. doi:10.1038/srep44804

16. Gong S, Yan Z, Liu Z, et al. Intestinal Microbiota Mediates the Susceptibility to Polymicrobial Sepsis-Induced Liver Injury by Granisetron Generation in Mice. *Hepatology*. Apr 2019;69(4):1751-1767. doi:10.1002/hep.30361

17. Tsoi H, Chu ESH, Zhang X, et al. Peptostreptococcus anaerobius Induces Intracellular Cholesterol Biosynthesis in Colon Cells to Induce Proliferation and Causes Dysplasia in Mice. *Gastroenterology*. May 2017;152(6):1419-1433 e5. doi:10.1053/j.gastro.2017.01.009

18. Wang X, Cui DN, Dai XM, et al. HuangQin Decoction Attenuates CPT-11-Induced Gastrointestinal Toxicity by Regulating Bile Acids Metabolism Homeostasis. *Front Pharmacol*. 2017;8:156. doi:10.3389/fphar.2017.00156

19. Song Z, Cai Y, Lao X, et al. Taxonomic profiling and populational patterns of bacterial bile salt hydrolase (BSH) genes based on worldwide human gut microbiome. *Microbiome*. Jan 23 2019;7(1):9. doi:10.1186/s40168-019-0628-3

20. Fiorucci S, Distrutti E. Bile Acid-Activated Receptors, Intestinal Microbiota, and the Treatment of Metabolic Disorders. *Trends Mol Med*. Nov 2015;21(11):702-714. doi:10.1016/j.molmed.2015.09.001

21. Macpherson AJ, Heikenwalder M, Ganal-Vonarburg SC. The Liver at the Nexus of Host-Microbial Interactions. *Cell Host Microbe*. Nov 9 2016;20(5):561-571. doi:10.1016/j.chom.2016.10.016
